# Supplementary material for: Using mobile health technology and community health workers to identify and refer caesarean-related surgical site infections in rural Rwanda: a randomised controlled trial protocol
Source: BMJ Open. 2018 May 8;8(5):e022214. doi: 10.1136/bmjopen-2018-022214 (PMC5942430; doi:10.1136/bmjopen-2018-022214)
Supplement: Supplementary data [file bmjopen-2018-022214supp001.pdf]

**Appendix.** Consent Form (English Version)

## INFORMED CONSENT FORM

This Informed Consent Form is for women 18 years of age and above who attend Kirehe District Hospital and receive cesarean section surgery. You are invited to participate in research on follow up of patients with surgical site infections post operation using mobile phones.

**The title of our research project is**: Using mHealth technology to identify and refer surgical site infections in Rwanda

**Principal Investigators (PIs):**


Robert Riviello
Rwanda Human Resources for Health Program, Harvard Medical School, Brigham and Women's Hospital
robertriviello@gmail.com +250783002502

Bethany Hedt-Gauthier
Partners in Health and Harvard Medical School
bethhedt@gmail.com +18572251945

Fred Kateera
Partners In Health
fkateera@partnersinhealth.onmicrosoft.com +250784684871


This consent form will give you the information you will need to understand why this study is being done and why you are being invited to participate. It also describes what you will need to do to participate. We encourage you to ask questions at any time. If you decide to participate, you will be asked to sign this form and I will keep it as a record of your agreement to participate. I will gladly provide you with a copy of this form to keep for your records upon your request.

**PURPOSE AND BACKGROUND**

Surgical site infections (SSI) represent a major source of morbidity and mortality worldwide and are disproportionately felt in low- and middle-income countries. You are invited to participate in a research study to assess the impact of the mobileHealth-supported delivery of the screening protocol by surgical CHWs on the rate of return to care of patients with SSI ten days post-operative. For patients who return, we will assess the severity of SSI at return to care. We aim to investigate timely and appropriate return to care of patients with SSIs in Rwanda, improving patient outcomes and reducing healthcare costs.

**PROCEDURES**

If you agree to participate in the study, you will be randomized into one of three study arms – Arm 1: home visit from the sCHW with screening using the mHealth tool; Arm 2: screening by sCHW over the phone using the mHealth tool; and Arm 3: standard of care, with no special contact from the sCHW or interaction with the mHealth tool. You will be instructed to return to

your local health center as soon as any of the signs of infection present. The study team will record basic demographic and clinical data.

If you are randomized into Arm 1 or 2, we will ask for addresses/phone numbers and availability to allow for follow-up by the sCHW. If you are randomized to Arm 1, sCHWs will visit you at ten post-operative days (±3 days) at the address provided. The sCHW will be assisted by a local village CHW to identify your home. Once there, the sCHW will administer the SSI screening protocol. A picture of your wound and GPS coordinates for your location will be taken. If you are randomized to Arm 2, you will be called by the sCHW on the tenth post-operative day (±3 days). The sCHW will administer the SSI screening protocol over the phone, prompted by the mHealth tool to ask the appropriate questions. If you are identified as having an SSI, the sCHW will ask you to go to your health center for care and from there you can be referred to KDH if necessary. If you are not identified to have an SSI, you will be reminded of the warning signs and follow-up instructions.

If you are randomized to Arm 3, you will receive standard of care, which is information upon your discharge about the signs of SSI. You will not receive any follow up from the sCHWs. You will be advised to return to your regional health center if any of the signs of an SSI do occur.

**PARTICIPANT SELECTION**
We are inviting all adults of 18 years and above who attend Kirehe District Hospital and receive cesarean section surgery to participate in this study.

**RISKS**
You will receive standard of care advice on surgical follow-up and when to return to care. If you are randomized into an arm where you have contact with a sCHW (Arms 1 and 2), you will be referred back to care if evidence of an SSI is present or will otherwise be reminded of advice on when to return to care. It is possible that the sCHW will give the wrong SSI diagnosis or that a patient may delay return to care because of an expected visit from an sCHW. This risk is moderate as the SSI screening protocol will have been tested for accuracy. However, this risk will be monitored.

**BENEFITS**
If you are randomized to Arms 1 or 2, you will have additional contact with a health care provider (sCHW) beyond the standard of care, which may lead to a more timely diagnosis of SSI. This may lead to an earlier presentation to care for appropriate treatment. You may also benefit from decreased barriers to follow-up care. Your participation may also help design quality improvement interventions that have the potential to directly affect the quality and efficiency of surgical care at KDH and other hospitals in Rwanda.

**EXTENT OF CONFIDENTIALITY**
Participation in research may involve a loss of privacy; however, your records will be handled as confidentially as possible. We will not be sharing the identity or information of those participating in the research. Information we collect from this research will be kept confidential and no one but the study staff will be able to see it. Your name will not be used in any written reports or publications that result from this research. Any information about you will have a

unique study number on it instead of your name. Only the study staff will know the number and we will lock that information up with a lock and key. Data will be kept for three years after the study is complete and then destroyed, per United States federal regulations.

**PAYMENT**
You will not receive any monetary compensation for participation in this study.

**QUESTIONS**
If you have any questions or concerns about your participation in this study, you should first contact the principal investigators at +250784684871 or bethhedt@gmail.com or robertriviello@gmail.com. If you have questions about your rights as a research participant, you may contact the Partners Healthcare Institutional Review Board (IRB), which is concerned with the protection of volunteers in research projects. You may reach the board office by calling +1 (617) 424-4100, or by emailing IRB@partners.org. Responses will be provided in one business day.

**PARTICIPATION IS VOLUNTARY**
You do not have to participate in this study if you do not want to. If you volunteer to be in this study, you may withdraw from it at any time without consequences of any kind or loss of benefits to which you are otherwise entitled. Whether you choose to participate or not does not impact the standard of care you receive from Kirehe District Hospital.

**DOCUMENTATION OF CONSENT**
I have read the information in this Informed Consent Form, or it has been read to me. Its general purposes, the particulars of involvement and possible risks, including the questions I have asked, have been explained to my satisfaction. I understand the information in this form and I have decided that I will participate in the research project described above. I understand I can withdraw at any time.

______________________________ ______________________________________

**Printed Name** of Study Participant **Signature** of Study Participant Date

______________________________ ____________

Signature of Person Obtaining Consent Date

**If the participant cannot read or write:**

I have witnessed the accurate reading of this Informed Consent Form to the potential participant, and the individual has had the opportunity to ask questions. I confirm that the individual has given consent freely.

**Print name** of witness______________________ **AND** **Thumb print** of participant

**Signature** of witness ______________________

Date ______________________
