# Supplementary material for: Using mobile health technology and community health workers to identify and refer caesarean-related surgical site infections in rural Rwanda: a randomised controlled trial protocol
Source: BMJ Open. 2018 May 8;8(5):e022214. doi: 10.1136/bmjopen-2018-022214 (PMC5942430; doi:10.1136/bmjopen-2018-022214)
Supplement: Author's manuscript [file bmjopen-2018-022214.draft_revisions.pdf]

BMJ Open is committed to open peer review. As part of this commitment we make the peer review history of every article we publish publicly available.

When an article is published we post the peer reviewers' comments and the authors' responses online. We also post the versions of the paper that were used during peer review. These are the versions that the peer review comments apply to.

The versions of the paper that follow are the versions that were submitted during the peer review process. They are not the versions of record or the final published versions. They should not be cited or distributed as the published version of this manuscript.

BMJ Open is an open access journal and the full, final, typeset and author-corrected version of record of the manuscript is available on our site with no access controls, subscription charges or pay-per-view fees (http://bmjopen.bmj.com).

If you have any questions on BMJ Open's open peer review process please email info.bmjopen@bmj.com

BMJ Open

# Using mobile health technology and community health workers to identify and refer cesarean-related surgical site infections in rural Rwanda: A randomized-control trial protocol



| | |
|---|---|
| Journal: | *BMJ Open* |
| Manuscript ID | bmjopen-2018-022214 |
| Article Type: | Protocol |
| Date Submitted by the Author: | 06-Feb-2018 |



| | |
|---|---|
| Complete List of Authors: | Sonderman, Kristin; Brigham and Women's Hospital, Center for Surgery and Public Health; Harvard Medical School, Global Health and Social Medicine<br>Nkurunziza, Theoneste; Partners in Health<br>Kateera, Fredrick; Partners in Health<br>Gruendl, Magdalena; Harvard Medical School, Global Health and Social Medicine<br>Koch, Rachel; Harvard Medical School, Global Health and Social Medicine<br>Gaju, Erick; Ministry of Health<br>Habiyakare, Caste; Ministry of Health<br>Matousek, Alexi; Brigham and Women's Hospital, Center for Surgery and Public Health<br>Nahimana, Evrard; Partners in Health<br>Ntakiyiruta, Georges; Ejo Heza Surgical Centre<br>Riviello, Robert; Brigham and Women's Hospital, Center for Surgery and Public Health; Harvard Medical School, Global Health and Social Medicine<br>Hedt-Gauthier, Bethany; Harvard Medical School, Global Health and Social Medicine |



| | |
|---|---|
| Keywords: | SURGERY, Mobile Health, Surgical site infections |


SCHOLARONE™ Manuscripts

# Using mobile health technology and community health workers to identify and refer cesarean-related surgical site infections in rural Rwanda: A randomized-control trial protocol

Kristin A. Sonderman, MD, MPH [A, B] Theoneste Nkurunziza, MS,[C] Fredrick Kateera, MD, PhD,[C] Magdalena Gruendl, MPH,[B] Rachel Koch, MD,[B] Erick Gaju,[D] Caste Habiyakare,[D] Alexi Matousek MD, MPH,[A] Evrard Nahimana, MD,[C] Georges Ntakiyiruta, MD,[E] Robert Riviello, MD, MPH,[A,B] Bethany Hedt-Gauthier, PhD[B,C]

Version 1
Feb 2 2018

For peer review only

**Affiliations:**
[A] Center for Surgery and Public Health, Brigham and Women's Hospital, Boston, MA
[B] Department of Global Health and Social Medicine, Harvard Medical School, Boston, MA
[C] Partners In Health, Kigali, Rwanda
[D] Ministry of Health, Kigali, Rwanda
[E] Ejo Heza Surgical Centre, Kigali, Rwanda

**Address Correspondence to:**
Kristin Sonderman MD, MPH
Center for Surgery and Public Health
Brigham and Women's Hospital
1620 Tremont Street
Boston, MA 02115
Work: 617.525.9281
Email: ksonderman@bwh.harvard.edu

**Manuscript Word Count:** 3348 / 4000
**Trial Registration Number:** NCT03311399

**ABSTRACT**

**Introduction:** Surgical site infections (SSIs) are a significant cause of morbidity and mortality in low- and middle-income countries, where rates of SSIs can reach 30%. Due to limited access, there is minimal follow up post-operatively. Community health workers (CHWs) have not yet been utilized for surgical patients in most settings. Advancements in telecommunication create an opportunity for mobile health (mHealth) tools to support CHWs. We aim to evaluate the use of mHealth technology to aid CHWs in identification of SSIs and promote referral of patients back to health care facilities.

**Methods and Analysis:** Prospective randomized control trial conducted at Kirehe District Hospital, Rwanda, from November 2017 - November 2018. Patients ≥18 years who undergo cesarean section are eligible. Non-residents of Kirehe District or patients who remain in hospital > 10 days postoperatively will be excluded. Patients will be randomized to one of three arms. For Arm 1, a CHW will visit the patient's home on postoperative day 10 (+/- 3 days) to administer an SSI screening protocol (fever, pain, or purulent drainage) using an electronic tablet. For Arm 2, the CHW will administer the screening protocol over the phone. For both Arms 1 and 2, the CHW will refer patients that respond "yes" to any of the questions to a health facility. For Arm 3, patients will not receive follow-up care. Our primary outcome will be the impact of the mHealth-CHW intervention on the rate of return to care for patients with an SSI.

**Ethics and Dissemination:** The study has received ethical approval from the Rwandan National Ethics Committee and Partners Healthcare. Results will be disseminated to Kirehe District Hospital, Rwanda Ministry of Health, Rwanda Surgical Society, Partners In Health, through conferences, and peer reviewed publications.

**Word Count:** 288/300



For peer review only - http://bmjopen.bmj.com/site/about/guidelines.xhtml

**STRENGTHS AND LIMITATIONS OF STUDY:**

- The greatest strength is that this is a prospective randomized control trial to most effectively evaluate the impact of a mobile health and CHW intervention on return to care following surgery.
- The screening protocol utilized has been previously validated by the study team in this setting.
- The study is well-resourced with significant on the ground logistical support through Partners in Health and the staff at Kirehe District Hospital.
- In addition to assessing the impact on patient return-to-care behaviors, this study will also allow us to describe the feasibility of mHealth and CHW interventions in this setting, beyond surgical interventions.
- Since validating the presence or absence of postoperative infections would interfere with the study aims, we can only compare the proportion of all patients that return to care with confirmed infections and must assume that the infection rates across arms are constant.



For peer review only - http://bmjopen.bmj.com/site/about/guidelines.xhtml

# INTRODUCTION

## Background

Surgical site infections (SSIs) are a major source of morbidity and mortality worldwide and the leading health-care-associated infection in the developing world.[1] The burden is disproportionately felt in low- and middle-income countries (LMICs), and especially by those in Africa where the rates of post-operative SSIs have been documented as high as 30.9%.[2] In these settings, SSIs often develop after patients are discharged home, and geographic and resource barriers prevent patients from routine postoperative follow-up.[3,4] In many LMICs, including Rwanda, follow-up with a surgical care provider after a procedure is not routine. Even when scheduled, rates of follow-up are low. A study from Central African Republic reported only 25% of surgical patients returned for their scheduled 30-day post-operative visit.[5] For patients who develop an SSI, failure to return or a delayed return to care is linked with poor health outcomes including sepsis, need for re-operation, death, and increased healthcare costs.[6]

In many LMICs, community health workers (CHWs) play a major role in delivering household-based care to vulnerable populations who might otherwise be unable to access health facilities.[7,8] Globally, the range of responsibilities of CHWs vary by program, whether polyvalent or topic-focused, such as the maternal and child health CHWs in Rwanda.[9] Regardless of the range, the number of responsibilities is typically high-leading to CHW work overload. Additional activities require extensive pre- or post-service training or provision of activity support aides. Recent advances in telecommunication and increasing access to mobile phones in LMICs create opportunities to use mobile health (mHealth) strategies to support CHWs. In Rwanda, 63% of the population in 2014 reportedly owned a cell phone, with 99% having access to mobile networks.[10] Multiple studies have shown that real-time use of



For peer review only - http://bmjopen.bmj.com/site/about/guidelines.xhtml

mHealth technologies increases adherence to health protocols in rural Africa,[11–14],[15] and also improves the perceived quality of care.[16]

In 2014, members of the study team carried out a pilot study in Haiti that involved CHWs following up with surgical patients once discharged and evaluating their wounds for an SSI.[17] The CHWs used an mHealth application that prompted the CHW to evaluate the wound for certain characteristics pertaining to SSIs as well as to take a photograph of the wound. The CHW's assessment of the wounds were then compared to a surgeon's assessment (using the photograph), and found 85% agreement. In the phase one study precluding this manuscript, over a 4-month period in 2017 (March- July) at KDH, we evaluated C-section patients at post-operative day (POD) 10 (+/- 3 days) and found a 10.3% SSI incidence (results yet to be published). In this study, we draw from lessons learned in the pilot to rigorously explore the use of mHealth-CHW interventions for postoperative follow-up of patients delivering via cesarean sections in rural Rwanda.

**Aims**

The overall study aim is to examine whether CHWs, guided by an mHealth-delivered screening protocol, can improve the identification of SSIs and inform a timely return to care among patients who undergo cesarean sections.

Specific objectives:

- Objective 1: Evaluate the impact of the mHealth-CHW interventions on patients returning to the health center or hospital for a possible SSI.
- Objective 2: To assess the feasibility of an mHealth-CHW intervention for post-operative follow-up.



For peer review only - http://bmjopen.bmj.com/site/about/guidelines.xhtml

After receipt of a voluntary written consent, enrolled patients will be randomly assigned to one of three arms (Figure 1):

- Arm 1– an mHealth-CHW intervention where the CHW visits the patient postoperatively, administers the screening protocol and refers the patient back to care if there is evidence of an SSI;
- Arm 2 – an mHealth-CHW intervention where the CHW calls the patient postoperatively, administers the screening protocol over the phone and refers the patient back to care if there is evidence of an SSI; or
- Arm 3 – standard of care (no routine follow up).

## METHODS AND ANALYSIS

### Study Location

This study will take place between November 2017 – November 2018 at Kirehe District Hospital (KDH)- one of 42 district hospitals in Rwanda. KDH is a Level 1 hospital, with 235 beds, operated by the Rwanda Ministry of Health and supported by the medical non-profit organization Partners In Health (PIH). The hospital serves a catchment area of 368,950 people, primarily residing in rural, outlying villages. KDH performs around 1,400 surgical operations a year, with the majority being cesarean sections (C-sections).[18] Nearly all C-sections are performed by general practitioner (GP) physicians, with occasional surgeries performed by visiting obstetricians.

### Study Population

This study will only include patients undergoing C-section delivery, which are the majority of



For peer review only - http://bmjopen.bmj.com/site/about/guidelines.xhtml

patients undergoing an operation, at KDH. Over 60% of all surgeries performed at Rwandan district hospitals are obstetric related.[19] All patients 18 years or older undergoing a C-section at KDH during a 12 month study enrollment window will be eligible for inclusion. Participants must be residents of Kirehe District. We will exclude patients who remain inpatient after POD 10 as the window for follow up we are interested in would have passed (10 days postoperative +/- 3 days). We will also exclude patients who are residents of Mahama refugee camp in Kirehe as the refugee camp is temporary and the patients are not covered by the existing CHW network.

**The SSI screening protocol**

The study SSI screening protocol will consist of three screening questions, which were developed and validated during Phase 1 of this study. Phase 1 was also carried out at KDH, and the three questions were selected to have the highest sensitivity while maintaining reasonable specificity for diagnosing an SSI. The optimization occurred over a 4-month period in 2017 and included post C-section surgical discharged patients 18 years or older. Patients returned to the hospital for evaluation on POD 10 (+/-3 days) and were evaluated by a general practitioner (GP). A CHW administered a 10-question SSI screening protocol assessing for: 1) increased pain since discharge; 2) fever since discharge, 3) erythema, 4) edema, 5) induration, 6) dehiscence, 7) drainage from the wound, 8) drainage with discoloration, 9) drainage with a foul odor, and 10) drainage with pus (purulent drainage). Using the GP's SSI diagnosis as the gold standard, we identified the following three questions as most sensitive and specific for SSI diagnosis: purulent drainage, pain and fever (Table 1).



For peer review only - http://bmjopen.bmj.com/site/about/guidelines.xhtml

**Table 1.** SSI Screening Protocol

| Question | Answer |
|---|---|
| Have you had a fever since discharge? | Yes/No |
| At the incision, have you had increasing pain? | Yes/ No |
| Any active drainage? | Yes/No |
| - What color is the fluid? | Brown, yellow, green or white / Red, pink, clear |

For peer review only

**Study Interventions**

The study involves two differ1ent interventions: use of mHealth and CHWs arms. For both interventions, patients will be screened at POD 10 (+/- 3 days). We selected this window because the majority of SSIs develop between POD 5-10 days and timely identification of SSIs is a critical aspect of the intervention.[20] In Arm 1, a CHW will travel to the patient's home to evaluate the patient. Prior to the visit, the patient will be called to confirm location and time. The hired surgical CHW will contact the local CHW who will guide the surgical CHW to the patient's home. Once at the patient's home, the local CHW will leave, and the surgical CHW will evaluate the patient using the SSI screening protocol administered on an electronic tablet and take a photo of the wound. In Arm 2, a CHW will call the patient on the phone on POD 10 (+/- 3 days). Three attempts will be made to reach the patient. The CHW will administer the screening protocol over the phone, prompted by the tablet application to ask the appropriate questions. For both intervention arms, if the patient answers yes to any of the three questions, the patient will be instructed by the CHW to present to the local health center for evaluation and referral to KDH if necessary. Patients not identified with an SSI will be reminded of proper wound care, warning signs of SSI and to follow-up should there be any change. In Arm 3,



For peer review only - http://bmjopen.bmj.com/site/about/guidelines.xhtml

patients will be given discharge instructions however will not have any contact with a CHW following discharge and therefore will serve as a control group.

**Study Consent, Enrollment, Randomization and Follow-up**

On POD 2, eligible patients will be identified. Study staff will read the consent form (appendix) to the patient in Kinyarwanda and solicit a signed consent. Once the patient is enrolled, there will be no special retention strategies as this will interfere with the overall study outcomes.

At discharge, the enrolled patients will be randomized to one of the three study arms described above. Study staff will prepare study packets, in sealed envelopes numbered consecutively. REDCap application will be used to randomly generate arm assignments to each packet. The assignment is independent of any patient factors, including whether the patient has access to a cell phone or lives in an area with cell phone coverage. In addition to the random arm assignment, the packet will include details on arm-specific follow-up such as follow-up plan for home visits (Arm 1) or phone call date (Arm 2). The packet will also include general discharge instructions including signs of a surgical site infection, how to contact study staff, and how to return to a health center for care or referral to KDH if a SSI is suspected by CHW.

All enrolled patients will be followed for 30 days post-operatively. If a patient is identified as having an SSI, she will be followed up to 90 days to document the progression and treatment of the infection. On POD 30, all patients will be called by a member of the study team to check in to see if they have returned to care. Study participants who return to care will be recorded by the register at the health facility where she presents (health center or hospital), and the study team will have regular check-ins with the register to obtain the list of patients who returned to care. Clinical data from those follow up visits will then be transcribed into REDCap



For peer review only - http://bmjopen.bmj.com/site/about/guidelines.xhtml

for each patient.

**Data Collection and Variables**

All study data will be collected, managed and store using REDCap electronic data capture tools hosted by Brigham and Women's Hospital. REDCap is a secure web application that can support both online or offline data collection for research studies.[21],[22] The REDCap mobile application will be utilized by CHWs to administer the SSI screening protocol. There will be five distinct time points of data collection. Study coordinators will have access to data to evaluate for completeness.

First, upon enrollment, all patients will provide basic demographics, socioeconomic and location data including but not limited to age, occupation, education, household income, insurance, home location, travel distance from the patient's home, patient's home village, cell, sector name, name of local CHW, phone number of the patient, phone number of a family member or a neighbor (in case the patient does not have personal phone), with permissions to call these numbers as part of follow-up. Secondly, on discharge, data collectors will complete a clinical chart review, extracting details on patient's past medical history, intraoperative data (pre-operative antibiotics, wound class, intraoperative complications), and post-operative care. Thirdly, for patients in Arms 1 and Arms 2, we will collect the responses of the SSI screening protocol. The CHW will click on the patient's ID number in REDCap, and the application will prompt the CHW to ask the three SSI screening protocol questions. The CHW will answer the questions on the tablet and the data will be stored. The fourth round will include the CHW separately collecting data on process indicators related to the implementation of the intervention. For Arm 1, these indicators will include: ability to visit the patient on the scheduled date, ability



For peer review only - http://bmjopen.bmj.com/site/about/guidelines.xhtml

to find the local CHW and the patient's home, travel time, presence of the patient at time of visit, willingness of the patient to allow CHW into home, patient compliance with the SSI screening protocol, if the patient allowed the CHW to perform an examination/ take a photo of the wound, and if there were any technical difficulties with the tablet or software. For Arm 2, these indicators will include: whether the patient was reached by phone, how many attempts were made, which number was called and who answered, total call time, and whether patient allowed the CHW to administer the SSI screening protocol. Finally, we will track the patient's return to care within 30 days post-operatively using a register posted at each of the 16 district health centers where staff can record any study patients who present to that location for care. The head of maternity at each health center will be a point person for this follow up register. The study coordinator will call each point person to check if a C-section patient showed up at any health center. If so, the study coordinator will visit the health centers that patients returned to. During the visit, the study coordinator will refer to the follow-up register to record into REDCap which date the patient returned, wound status, diagnosis, treatment provided, and if they were referred to KDH for further care. There will be a similar patient tracker log in the maternity ward reception at KDH to document patients referred to the hospital. This log will be completed by the reception nurse who will notify the study data collector who will input to REDCap. Finally, all patients with phone numbers provided will be called on POD 30 to inquire about any re-admissions or visits to other healthcare facilities. Study staff will extract from the clinical chart the presence of an SSI, severity, treatment obtained, need for operative intervention, hospitalization, and/ or complications.

**Analyses**



For peer review only - http://bmjopen.bmj.com/site/about/guidelines.xhtml

All analyses will be completed as intention to treat. For objective 1, the primary outcome is whether a patient returns to care at a health center or district hospital with a provider-confirmed SSI. We will compare the proportion of patients who returned for follow-up with an SSI in Arms 1 and 2 to Arm 3 using a two-sided, two-sample test of proportions at the $\alpha$=0.05 significance level. The analyses assume that the rates of true SSIs are constant across the three arms, but that the proportion of these infections that return to care will vary across the study arms as a result of the intervention. We have purposely chosen not to trace patients to establish their true SSI status, as this would interfere with care seeking behavior. However, we will perform a sensitivity analysis (changing the null hypothesis from $p_1=p_2$ to $p_1=kp_2$, where $k$ reflects differences in SSI rates) to determine under what range of SSI rates the results are still valid. As a secondary outcome for objective 1, we will look at time to return-to-care for patients with SSI dichotomized as within 15 PODs or more than 15 PODs. We will use a logistic regression model to assess the impact of study arm on timely return to care, controlling for potential confounders collected at enrollment. For objective 2, we will assess the implementation feasibility of the CHW-mHealth intervention by quantifying intervention indicators. For each indicator, we will report the percent of eligible encounters for which that step was successfully completed, and will categorize a specific component as feasible if at least 85% of eligible counters have that step completed. For Arms 1 and 2, we will calculate a comprehensive feasibility measure that will assess the percent of encounters that successfully implemented the full intervention, which we aim to achieve with at least 85% of patient encounters.

**Power calculation**

Over the 12-month study period, we expect 78 patients/month or 1092 patients total to be eligible



For peer review only - http://bmjopen.bmj.com/site/about/guidelines.xhtml

for inclusion. Assuming a 1-1-1 randomization, 26 patients/month or 364 patients total will be randomized to each arm (Table 2).

**Table 2.** Sample Size Calculation

| | **Arm 1:** Home visit + Protocol | **Arm 2:** Phone call + Protocol | **Arm 3:** Standard of care |
|---|---|---|---|
| Total Patients | 364 | 364 | 364 |
| Anticipated SSIs | 55 | 55 | 55 |
| Hypothesized patients to return with SSIs | 44 (80%) | 44 (80%) | 22 (40%) |
| Overall hypothesized proportion that will return with SSI | 0.12 | 0.12 | 0.06 |

We assume a constant SSI rate across the three arms of 15% (based on data from preliminary chart reviews referenced above). We assume more patients with SSIs will return to care in Arms 1 and 2 compared to Arm 3 (80% of SSIs in Arms 1 and 2 compared to 40% in Arm 3). This corresponds to an overall return to care rate of 12% in Arms 1 and 2 and 6% in Arm 3. We would have an 81% power to detect a difference between the proportion of patients that returned with an SSI in Arms 1 and 2 (12%) as compared to Arm 3 (6%) with a two-sided test at the $\alpha$=0.05 significance level.

## ETHICS AND DISSEMINATION:

Study participants will be informed on the intent of the study, potential benefits and risks of their enrollment, and how these will be minimized. Those who wish to enroll will be informed of their right to withdraw throughout the study period. All data collectors will sign confidentiality training and agreements; study coordinators and CHWs. Risks to privacy will be minimized by



For peer review only

having all mobile devices and computers password protected. Data will be stored on HIPAA-compliant servers, and data will be de-identified prior to any analysis.

**Benefits and Risks**

The study does not alter the standard of care in any way and therefore there is minimal to no increased risk to the patient. Participants will likely benefit from this study in that the intervention we hypothesize will lead to a timelier diagnosis of SSI and will encourage patients to return to care. Patients enrolled in both Arms 1 and 2 will have additional contact with a health care provider (CHW) beyond the current standard of care. While not all participants may need this earlier screening, as not all will have surgical complications, the risks and discomforts associated with the screening are minimal. On a systems level, this study will benefit the local providers and research staff to understand whether CHWs can be used in this capacity for postoperative follow-up.

A potential risk will be decreasing the likelihood of a patient return to care when needed under the mHealth-CHW interventions. It is possible that the CHW will give the wrong SSI diagnosis or that a patient may delay return to care because of an expected visit from a CHW. This risk is moderate and will be monitored by a Data and Safety Monitoring Board (DSMB). Finally, a potential risk would be a breach in confidentiality, resulting in the disclosure of patient information. This risk is considered minimal as unique codes will be used in place of participant names throughout the study. Only PIs and study coordinators will have access to the final de-identified database.

**Data and Safety Monitoring Board (DSMB)**



For peer review only - http://bmjopen.bmj.com/site/about/guidelines.xhtml

The DSMB will be designated to oversee the safety and effectiveness of the study. This committee will include one global surgery expert, one Rwandan health practitioner and one statistician. Meetings of the DSMB will be held twice – once at the start of the study and 6 months after the start of Phase 2. At the first meeting the DSMB will discuss the protocol, suggest modifications, and establish guidelines to study monitoring by the Board.

At the second meeting, we will present the DSMB an interim analysis report, which will compare rates of return between the three study arms and include a list of adverse events of this study, if any. We anticipate ½ of the total cohort of patients will be included in this interim analysis. If the proportion who have returned in Arms 1 and 2 is significantly lower compared to standard of care, then the study will be stopped or one study arm will be dropped. Further, if there are significantly more complex cases at return (higher rates of readmission or reoperation) in Arms 1 or 2, then the study will be stopped or one study arm will be dropped. The outcome of the DSMB review will be summarized in a letter to the IRBs of all participating institutions. A recommendation by the DSMB to terminate the study would be communicated to the NIH Director, who will then accept or decline the recommendation.

**Ethics Approvals**

The study has received IRB approval both in the United States and in Rwanda. IRB approval in the United States has been achieved through Partners Healthcare (2016P001943/MGH). The Rwanda National Ethics Committee has reviewed and approved the study (848/RNEC/2016). Any proposed protocol amendments would undergo review and approvals by IRBs before further implementation.



For peer review only - http://bmjopen.bmj.com/site/about/guidelines.xhtml

**Dissemination**

Results will be disseminated to the staff at Kirehe District Hospital, the Rwanda Ministry of Health, including the electronic Health and CHW departments, the Rwanda Surgical Society, and PIH. Results will also be disseminated at regional and international conferences and via peer reviewed publications.

For peer review only



For peer review only - http://bmjopen.bmj.com/site/about/guidelines.xhtml

**AUTHORS CONTRIBUTIONS:** BHG, RR and FK are the three primary investigators for the study, instigated the original idea for the study, developed the funding proposal, and applied for funding. EG, CH, AM, EN, GN advised the study objectives and scientific content of this study. KAS, TN and RK are study coordinators and contributed to writing of the protocol. MG contributed to writing of the protocol and Figures/Tables. All authors read and commented on drafts, and approved the final version.

**NO AUTHORS HAD COMPETING INTERESTS**

**FUNDING/SPONSOR:** This work was supported by The National Institute of Health grant number NIH Grant 1R21EB022369 – 01. The funders have no role in the study design, collection, management, analysis, or interpretation of results. Contact info: Ruthann Rand, Grants Management Officer, email: Rudy.Rand@nih.hhs.gov

For peer review only



For peer review only - http://bmjopen.bmj.com/site/about/guidelines.xhtml

**Figure Legend**

Figure 1. Study Design

For peer review only



For peer review only - http://bmjopen.bmj.com/site/about/guidelines.xhtml

**Appendix.** Consent Form (English Version)

## INFORMED CONSENT FORM

This Informed Consent Form is for women 18 years of age and above who attend Kirehe District Hospital and receive cesarean section surgery. You are invited to participate in research on follow up of patients with surgical site infections post operation using mobile phones.

**The title of our research project is**: Using mHealth technology to identify and refer surgical site infections in Rwanda

**Principal Investigators (PIs):**

Robert Riviello
Rwanda Human Resources for Health Program, Harvard Medical School, Brigham and Women's Hospital
robertriviello@gmail.com +250783002502

Bethany Hedt-Gauthier
Partners in Health and Harvard Medical School
bethhedt@gmail.com +18572251945

Fred Kateera
Partners In Health
fkateera@partnersinhealth.onmicrosoft.com +250784684871

This consent form will give you the information you will need to understand why this study is being done and why you are being invited to participate. It also describes what you will need to do to participate. We encourage you to ask questions at any time. If you decide to participate, you will be asked to sign this form and I will keep it as a record of your agreement to participate. I will gladly provide you with a copy of this form to keep for your records upon your request.

**PURPOSE AND BACKGROUND**

Surgical site infections (SSI) represent a major source of morbidity and mortality worldwide and are disproportionately felt in low- and middle-income countries. You are invited to participate in a research study to assess the impact of the mobileHealth-supported delivery of the screening protocol by surgical CHWs on the rate of return to care of patients with SSI ten days post-operative. For patients who return, we will assess the severity of SSI at return to care. We aim to investigate timely and appropriate return to care of patients with SSIs in Rwanda, improving patient outcomes and reducing healthcare costs.

**PROCEDURES**

If you agree to participate in the study, you will be randomized into one of three study arms – Arm 1: home visit from the sCHW with screening using the mHealth tool; Arm 2: screening by sCHW over the phone using the mHealth tool; and Arm 3: standard of care, with no special contact from the sCHW or interaction with the mHealth tool. You will be instructed to return to



For peer review only - http://bmjopen.bmj.com/site/about/guidelines.xhtml

your local health center as soon as any of the signs of infection present. The study team will record basic demographic and clinical data.

If you are randomized into Arm 1 or 2, we will ask for addresses/phone numbers and availability to allow for follow-up by the sCHW. If you are randomized to Arm 1, sCHWs will visit you at ten post-operative days (±3 days) at the address provided. The sCHW will be assisted by a local village CHW to identify your home. Once there, the sCHW will administer the SSI screening protocol. A picture of your wound and GPS coordinates for your location will be taken. If you are randomized to Arm 2, you will be called by the sCHW on the tenth post-operative day (±3 days). The sCHW will administer the SSI screening protocol over the phone, prompted by the mHealth tool to ask the appropriate questions. If you are identified as having an SSI, the sCHW will ask you to go to your health center for care and from there you can be referred to KDH if necessary. If you are not identified to have an SSI, you will be reminded of the warning signs and follow-up instructions.

If you are randomized to Arm 3, you will receive standard of care, which is information upon your discharge about the signs of SSI. You will not receive any follow up from the sCHWs. You will be advised to return to your regional health center if any of the signs of an SSI do occur.

**PARTICIPANT SELECTION**
We are inviting all adults of 18 years and above who attend Kirehe District Hospital and receive cesarean section surgery to participate in this study.

**RISKS**
You will receive standard of care advice on surgical follow-up and when to return to care. If you are randomized into an arm where you have contact with a sCHW (Arms 1 and 2), you will be referred back to care if evidence of an SSI is present or will otherwise be reminded of advice on when to return to care. It is possible that the sCHW will give the wrong SSI diagnosis or that a patient may delay return to care because of an expected visit from an sCHW. This risk is moderate as the SSI screening protocol will have been tested for accuracy. However, this risk will be monitored.

**BENEFITS**
If you are randomized to Arms 1 or 2, you will have additional contact with a health care provider (sCHW) beyond the standard of care, which may lead to a more timely diagnosis of SSI. This may lead to an earlier presentation to care for appropriate treatment. You may also benefit from decreased barriers to follow-up care. Your participation may also help design quality improvement interventions that have the potential to directly affect the quality and efficiency of surgical care at KDH and other hospitals in Rwanda.

**EXTENT OF CONFIDENTIALITY**
Participation in research may involve a loss of privacy; however, your records will be handled as confidentially as possible. We will not be sharing the identity or information of those participating in the research. Information we collect from this research will be kept confidential and no one but the study staff will be able to see it. Your name will not be used in any written reports or publications that result from this research. Any information about you will have a



For peer review only - http://bmjopen.bmj.com/site/about/guidelines.xhtml

unique study number on it instead of your name. Only the study staff will know the number and we will lock that information up with a lock and key. Data will be kept for three years after the study is complete and then destroyed, per United States federal regulations.

**PAYMENT**

You will not receive any monetary compensation for participation in this study.

**QUESTIONS**

If you have any questions or concerns about your participation in this study, you should first contact the principal investigators at +250784684871 or bethhedt@gmail.com or robertriviello@gmail.com. If you have questions about your rights as a research participant, you may contact the Partners Healthcare Institutional Review Board (IRB), which is concerned with the protection of volunteers in research projects. You may reach the board office by calling +1 (617) 424-4100, or by emailing IRB@partners.org. Responses will be provided in one business day.

**PARTICIPATION IS VOLUNTARY**

You do not have to participate in this study if you do not want to. If you volunteer to be in this study, you may withdraw from it at any time without consequences of any kind or loss of benefits to which you are otherwise entitled. Whether you choose to participate or not does not impact the standard of care you receive from Kirehe District Hospital.

**DOCUMENTATION OF CONSENT**

I have read the information in this Informed Consent Form, or it has been read to me. Its general purposes, the particulars of involvement and possible risks, including the questions I have asked, have been explained to my satisfaction. I understand the information in this form and I have decided that I will participate in the research project described above. I understand I can withdraw at any time.

**Printed Name** of Study Participant **Signature** of Study Participant Date

Signature of Person Obtaining Consent Date

**If the participant cannot read or write:**



For peer review only - http://bmjopen.bmj.com/site/about/guidelines.xhtml

I have witnessed the accurate reading of this Informed Consent Form to the potential participant, and the individual has had the opportunity to ask questions. I confirm that the individual has given consent freely.

**Print name** of witness______________________ **AND** **Thumb print** of participant

**Signature** of witness _______________________

Date _______________________



For peer review only - http://bmjopen.bmj.com/site/about/guidelines.xhtml

## REFERENCES


1 Allegranzi B, Bagheri Nejad S, Combescure C, *et al.* Burden of endemic health-care-associated infection in developing countries: systematic review and meta-analysis. *Lancet (London, England)* 2011;**377**:228–41. doi:10.1016/S0140-6736(10)61458-4

2 Nejad SB, Allegranzi B, Syed S, *et al.* Health-care-associated infection in Africa: a systematic review. *Bull World Health Organ* 2011;**89**:757–65. doi:10.2471/BLT.11.088179

3 Grimes CE, Bowman KG, Dodgion CM, *et al.* Systematic Review of Barriers to Surgical Care in Low-Income and Middle-Income Countries. *World J Surg* 2011;**35**:941–50. doi:10.1007/s00268-011-1010-1

4 Hsia RY, Mbembati NA, Macfarlane S, *et al.* Access to emergency and surgical care in sub-Saharan Africa: the infrastructure gap. *Health Policy Plan* 2012;**27**:234–44. doi:10.1093/heapol/czr023

5 Bercion R, Gaudeuille A, Mapouka PA, *et al.* [Surgical site infection survey in the orthopaedic surgery department of the "Hôpital communautaire de Bangui," Central African Republic]. *Bull Soc Pathol Exot* 2007;**100**:197–200.http://www.ncbi.nlm.nih.gov/pubmed/17824315 (accessed 10 Jun 2017).

6 Gottrup F, Melling A, Hollander D. An overview of surgical site infections: aetiology, incidence and risk factors. *EWMA J* Published Online First: 2005.http://www.worldwidewounds.com/2005/september/Gottrup/Surgical-Site-Infections-Overview.html (accessed 13 Jun 2017).

7 Dillip A, Kimatta S, Embrey M, *et al.* Can formalizing links among community health workers, accredited drug dispensing outlet dispensers, and health facility staff increase their collaboration to improve prompt access to maternal and child care? A qualitative study in Tanzania. *BMC Health Serv Res* 2017;**17**:416. doi:10.1186/s12913-017-2382-1

8 Asiimwe S, Ross JM, Arinaitwe A, *et al.* Expanding HIV testing and linkage to care in southwestern Uganda with community health extension workers. *J Int AIDS Soc* 2017;**20**:80–7. doi:10.7448/IAS.20.5.21633

9 Haver J, Brieger W, Zoungrana J, *et al.* Experiences engaging community health workers to provide maternal and newborn health services: Implementation of four programs. *Int J Gynecol Obstet* 2015;**130**:S32–9. doi:10.1016/j.ijgo.2015.03.006

10 *Statistical YearBook 2014 | National Institute of Statistics Rwanda*. Kigali: 2014. http://statistics.gov.rw/publication/statistical-yearbook-2016 (accessed 10 Jul 2017).

11 Mitchell M, Hedt-Gauthier B. Using electronic technology to improve clinical care–results from a before-after cluster trial to evaluate assessment and classification of sick children. *BMC Med* Published Online First: 2013.https://bmcmedinformdecismak.biomedcentral.com/articles/10.1186/1472-6947-13-95 (accessed 13 Jun 2017).

12 Ngabo F, Nguimfack J, Nwaigwe F, *et al.* Designing and Implementing an Innovative SMS-based alert system (RapidSMS-MCH) to monitor pregnancy and reduce maternal and child deaths in Rwanda. *Pan Afr Med J* 2012;**13**:31.http://www.ncbi.nlm.nih.gov/pubmed/23330022 (accessed 25 Aug 2017).

13 Elbur AI, MA Y, ElSayed ASA, *et al.* Post-discharge surveillance of wound infections by telephone calls method in a Sudanese Teaching Hospital. *J Infect Public Health* 2013;**6**:339–46. doi:10.1016/j.jiph.2013.04.005




For peer review only - http://bmjopen.bmj.com/site/about/guidelines.xhtml

14 Nguhuni B, De Nardo P, Gentilotti E, *et al.* Reliability and validity of using telephone calls for post-discharge surveillance of surgical site infection following caesarean section at a tertiary hospital in Tanzania. *Antimicrob Resist Infect Control* 2017;**6**:43. doi:10.1186/s13756-017-0205-0
15 Matousek A, Paik K, Winkler E, *et al.* Community health workers and smartphones for the detection of surgical site infections in rural Haiti: a pilot study. *Lancet* 2015;**385**:S47. doi:10.1016/S0140-6736(15)60842-X
16 Mitchell M, Getchell M, Nkaka M. Perceived improvement in integrated management of childhood illness implementation through use of mobile technology: qualitative evidence from a pilot study in. *J Heal* Published Online First: 2012.http://www.tandfonline.com/doi/abs/10.1080/10810730.2011.649105 (accessed 13 Jun 2017).
17 Matousek A, Addington S, Paik K. Community Health Workers for Surgery: A Pilot Study of an mHealth Application for the Early Detection of Surgical Site Infection in Rural Haiti. *J* Published Online First: 2014.https://www.journalacs.org/article/S1072-7515(14)00676-0/fulltext (accessed 13 Jun 2017).
18 Muhirwa E, Habiyakare C, Hedt-Gauthier BL, *et al.* Non-Obstetric Surgical Care at Three Rural District Hospitals in Rwanda: More Human Capacity and Surgical Equipment May Increase Operative Care. *World J Surg* 2016;**40**:2109–16. doi:10.1007/s00268-016-3515-0
19 Petroze RT, Nzayisenga a, Rusanganwa V, *et al.* Comprehensive national analysis of emergency and essential surgical capacity in Rwanda. *Br J Surg* 2012;**99**:436–43. doi:10.1002/bjs.7816
20 Health NCC for W and C. Surgical Site Infection: Prevention and Treatment of Surgical Site Infection. London: : RCOG Press 2008. https://www.ncbi.nlm.nih.gov/books/NBK53724/
21 Klipin M, Mare I, Hazelhurst S, *et al.* The process of installing REDCap, a web based database supporting biomedical research: the first year. *Appl Clin Inform* 2014;**5**:916–29. doi:10.4338/ACI-2014-06-CR-0054
22 Harris PA, Taylor R, Thielke R, *et al.* Research electronic data capture (REDCap)--a metadata-driven methodology and workflow process for providing translational research informatics support. *J Biomed Inform* 2009;**42**:377–81. doi:10.1016/j.jbi.2008.08.010

For peer review only

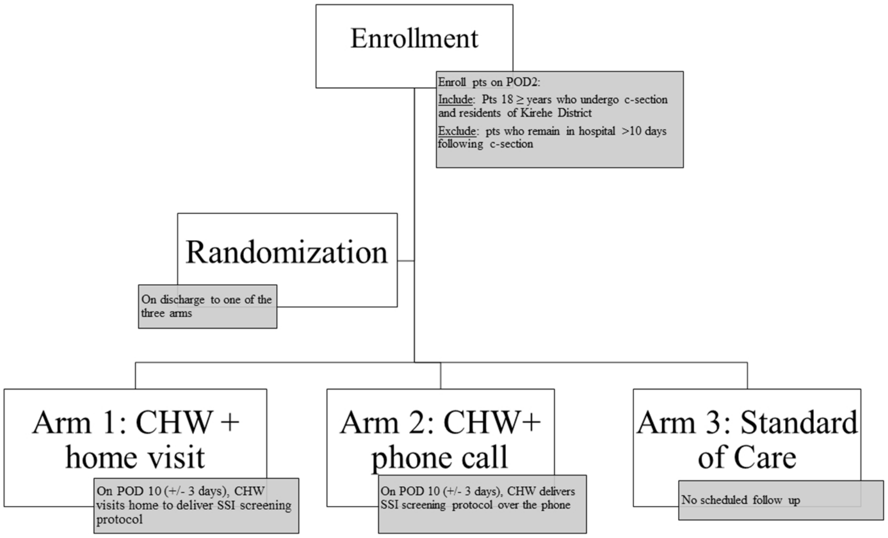


Study Design

338x190mm (96 x 96 DPI)

For peer review only - http://bmjopen.bmj.com/site/about/guidelines.xhtml

STANDARD PROTOCOL ITEMS: RECOMMENDATIONS FOR INTERVENTIONAL TRIALS

SPIRIT 2013 Checklist: Recommended items to address in a clinical trial protocol and related documents*

| Section/item | ItemNo/ Pg No | Description |
|---|---|---|
| **Administrative information** | | |
| Title | 1/ 1 | Descriptive title identifying the study design, population, interventions, and, if applicable, trial acronym |
| Trial registration | 2/ 1 | Trial identifier and registry name. If not yet registered, name of intended registry |
| | 2b/ yes, per below | All items from the World Health Organization Trial Registration Data Set |
| Protocol version | 3/ 1 | Date and version identifier |
| Funding | 4/17 | Sources and types of financial, material, and other support |
| Roles and responsibilities | 5a/ 1,17 | Names, affiliations, and roles of protocol contributors |
| | 5b/ 17 | Name and contact information for the trial sponsor |
| | 5c/ 17 | Role of study sponsor and funders, if any, in study design; collection, management, analysis, and interpretation of data; writing of the report; and the decision to submit the report for publication, including whether they will have ultimate authority over any of these activities |
| | 5d /14,15 | Composition, roles, and responsibilities of the coordinating centre, steering committee, endpoint adjudication committee, data management team, and other individuals or groups overseeing the trial, if applicable (see Item 21a for data monitoring committee) |
| **Introduction** | | |
| Background and rationale | 6a/ 4 | Description of research question and justification for undertaking the trial, including summary of relevant studies (published and unpublished) examining benefits and harms for each intervention |
| | 6b/ 6 | Explanation for choice of comparators |
| Objectives | 7/ 5,6 | Specific objectives or hypotheses |

For peer review only

| | | |
|---|---|---|
| Trial design | 8/ 8 | Description of trial design including type of trial (eg, parallel group, crossover, factorial, single group), allocation ratio, and framework (eg, superiority, equivalence, noninferiority, exploratory) |

**Methods: Participants, interventions, and outcomes**

| | | |
|---|---|---|
| Study setting | 9/ 6 | Description of study settings (eg, community clinic, academic hospital) and list of countries where data will be collected. Reference to where list of study sites can be obtained |
| Eligibility criteria | 10/ 7 | Inclusion and exclusion criteria for participants. If applicable, eligibility criteria for study centres and individuals who will perform the interventions (eg, surgeons, psychotherapists) |
| Interventions | 11a/ 8 | Interventions for each group with sufficient detail to allow replication, including how and when they will be administered |
| | 11b/ 14,15 | Criteria for discontinuing or modifying allocated interventions for a given trial participant (eg, drug dose change in response to harms, participant request, or improving/worsening disease) |
| | 11c/ 11 | Strategies to improve adherence to intervention protocols, and any procedures for monitoring adherence (eg, drug tablet return, laboratory tests) |
| | 11d/ NA | Relevant concomitant care and interventions that are permitted or prohibited during the trial |
| Outcomes | 12/ 12 | Primary, secondary, and other outcomes, including the specific measurement variable (eg, systolic blood pressure), analysis metric (eg, change from baseline, final value, time to event), method of aggregation (eg, median, proportion), and time point for each outcome. Explanation of the clinical relevance of chosen efficacy and harm outcomes is strongly recommended |
| Participant timeline | 13/ 9,10 | Time schedule of enrolment, interventions (including any run-ins and washouts), assessments, and visits for participants. A schematic diagram is highly recommended (see Figure) |
| Sample size | 14/ 13 | Estimated number of participants needed to achieve study objectives and how it was determined, including clinical and statistical assumptions supporting any sample size calculations |
| Recruitment | 15/ NA | Strategies for achieving adequate participant enrolment to reach target sample size |

**Methods: Assignment of interventions (for controlled trials)**

Allocation:

| | | |
|---|---|---|
| Sequence generation | 16a/ 9 | Method of generating the allocation sequence (eg, computer-generated random numbers), and list of any factors for stratification. To reduce predictability of a random sequence, details of any planned restriction (eg, blocking) should be provided in a separate document that is unavailable to those who enrol participants or assign interventions |
| Allocation concealment mechanism | 16b/ 9 | Mechanism of implementing the allocation sequence (eg, central telephone; sequentially numbered, opaque, sealed envelopes), describing any steps to conceal the sequence until interventions are assigned |
| Implementation | 16c/ 9 | Who will generate the allocation sequence, who will enrol participants, and who will assign participants to interventions |
| Blinding (masking) | 17a/ NA | Who will be blinded after assignment to interventions (eg, trial participants, care providers, outcome assessors, data analysts), and how |
| | 17b/ NA | If blinded, circumstances under which unblinding is permissible, and procedure for revealing a participant's allocated intervention during the trial |
| **Methods: Data collection, management, and analysis** | | |
| Data collection methods | 18a/ 10,11 | Plans for assessment and collection of outcome, baseline, and other trial data, including any related processes to promote data quality (eg, duplicate measurements, training of assessors) and a description of study instruments (eg, questionnaires, laboratory tests) along with their reliability and validity, if known. Reference to where data collection forms can be found, if not in the protocol |
| | 18b/ 11 | Plans to promote participant retention and complete follow-up, including list of any outcome data to be collected for participants who discontinue or deviate from intervention protocols |
| Data management | 19/ 13,14 | Plans for data entry, coding, security, and storage, including any related processes to promote data quality (eg, double data entry; range checks for data values). Reference to where details of data management procedures can be found, if not in the protocol |
| Statistical methods | 20a/ 11,12 | Statistical methods for analysing primary and secondary outcomes. Reference to where other details of the statistical analysis plan can be found, if not in the protocol |
| | 20b/ 11,12 | Methods for any additional analyses (eg, subgroup and adjusted analyses) |

| | | |
|---|---|---|
| | 20c/ 11,12 | Definition of analysis population relating to protocol non-adherence (eg, as randomised analysis), and any statistical methods to handle missing data (eg, multiple imputation) |
| **Methods: Monitoring** | | |
| Data monitoring | 21a/ 14,15 | Composition of data monitoring committee (DMC); summary of its role and reporting structure; statement of whether it is independent from the sponsor and competing interests; and reference to where further details about its charter can be found, if not in the protocol. Alternatively, an explanation of why a DMC is not needed |
| | 21b/ 15 | Description of any interim analyses and stopping guidelines, including who will have access to these interim results and make the final decision to terminate the trial |
| Harms | 22/ 14 | Plans for collecting, assessing, reporting, and managing solicited and spontaneously reported adverse events and other unintended effects of trial interventions or trial conduct |
| Auditing | 23/ NA | Frequency and procedures for auditing trial conduct, if any, and whether the process will be independent from investigators and the sponsor |
| **Ethics and dissemination** | | |
| Research ethics approval | 24/ 15 | Plans for seeking research ethics committee/institutional review board (REC/IRB) approval |
| Protocol amendments | 25/ 15 | Plans for communicating important protocol modifications (eg, changes to eligibility criteria, outcomes, analyses) to relevant parties (eg, investigators, REC/IRBs, trial participants, trial registries, journals, regulators) |
| Consent or assent | 26a/ 9 | Who will obtain informed consent or assent from potential trial participants or authorised surrogates, and how (see Item 32) |
| | 26b/ 9 | Additional consent provisions for collection and use of participant data and biological specimens in ancillary studies, if applicable |
| Confidentiality | 27/ 13,14 | How personal information about potential and enrolled participants will be collected, shared, and maintained in order to protect confidentiality before, during, and after the trial |
| Declaration of interests | 28/ 17 | Financial and other competing interests for principal investigators for the overall trial and each study site |
| Access to data | 29/ 14 | Statement of who will have access to the final trial dataset, and disclosure of contractual agreements that limit such access for investigators |

review only

| | | |
|---|---|---|
| Ancillary and post-trial care | 30/ NA | Provisions, if any, for ancillary and post-trial care, and for compensation to those who suffer harm from trial participation |
| Dissemination policy | 31a/ 16 | Plans for investigators and sponsor to communicate trial results to participants, healthcare professionals, the public, and other relevant groups (eg, via publication, reporting in results databases, or other data sharing arrangements), including any publication restrictions |
| | 31b/ NA | Authorship eligibility guidelines and any intended use of professional writers |
| | 31c/ NA | Plans, if any, for granting public access to the full protocol, participant-level dataset, and statistical code |
| **Appendices** | | |
| Informed consent materials | 32/ 19,20 | Model consent form and other related documentation given to participants and authorised surrogates |
| Biological specimens | 33/ NA | Plans for collection, laboratory evaluation, and storage of biological specimens for genetic or molecular analysis in the current trial and for future use in ancillary studies, if applicable |

*It is strongly recommended that this checklist be read in conjunction with the SPIRIT 2013 Explanation & Elaboration for important clarification on the items. Amendments to the protocol should be tracked and dated. The SPIRIT checklist is copyrighted by the SPIRIT Group under the Creative Commons "Attribution-NonCommercial-NoDerivs 3.0 Unported" license.

For peer review only

BMJ Open

# Using mobile health technology and community health workers to identify and refer cesarean-related surgical site infections in rural Rwanda: A randomized-control trial protocol

| | |
|---|---|
| Journal: | *BMJ Open* |
| Manuscript ID | bmjopen-2018-022214.R1 |
| Article Type: | Protocol |
| Date Submitted by the Author: | 29-Mar-2018 |
| Complete List of Authors: | Sonderman, Kristin; Brigham and Women's Hospital, Center for Surgery and Public Health; Harvard Medical School, Global Health and Social Medicine<br>Nkurunziza, Theoneste; Partners in Health<br>Kateera, Fredrick; Partners in Health<br>Gruendl, Magdalena; Harvard Medical School, Global Health and Social Medicine<br>Koch, Rachel; Harvard Medical School, Global Health and Social Medicine<br>Gaju, Erick; Ministry of Health<br>Habiyakare, Caste; Ministry of Health<br>Matousek, Alexi; Brigham and Women's Hospital, Center for Surgery and Public Health<br>Nahimana, Evrard; Partners in Health<br>Ntakiyiruta, Georges; Ejo Heza Surgical Centre<br>Riviello, Robert; Brigham and Women's Hospital, Center for Surgery and Public Health; Harvard Medical School, Global Health and Social Medicine<br>Hedt-Gauthier, Bethany; Harvard Medical School, Global Health and Social Medicine |
| <b>Primary Subject Heading</b>: | Surgery |
| Secondary Subject Heading: | Global health |
| Keywords: | SURGERY, Mobile Health, Surgical site infections |
| | |

SCHOLARONE™
Manuscripts

For peer review only - http://bmjopen.bmj.com/site/about/guidelines.xhtml

# Using mobile health technology and community health workers to identify and refer cesarean-related surgical site infections in rural Rwanda: A randomized-control trial protocol

Kristin A. Sonderman, MD, MPH [A, B] Theoneste Nkurunziza, MS,[C] Fredrick Kateera, MD, PhD,[C] Magdalena Gruendl, MPH,[B] Rachel Koch, MD,[B] Erick Gaju,[D] Caste Habiyakare,[D] Alexi Matousek MD, MPH,[A] Evrard Nahimana, MD,[C] Georges Ntakiyiruta, MD,[E] Robert Riviello, MD, MPH,[A,B] Bethany Hedt-Gauthier, PhD[B,C]

Version 3
March 27th 2018

For peer review only

**Affiliations:**
[A] Center for Surgery and Public Health, Brigham and Women's Hospital, Boston, MA
[B] Department of Global Health and Social Medicine, Harvard Medical School, Boston, MA
[C] Partners In Health, Kigali, Rwanda
[D] Ministry of Health, Kigali, Rwanda
[E] Ejo Heza Surgical Centre, Kigali, Rwanda

**Address Correspondence to:**
Kristin Sonderman MD, MPH
Center for Surgery and Public Health
Brigham and Women's Hospital
1620 Tremont Street
Boston, MA 02115
Work: 617.525.9281
Email: ksonderman@bwh.harvard.edu

**Manuscript Word Count:** 3756 / 4000
**Trial Registration Number:** NCT03311399

## ABSTRACT

**Introduction:** Surgical site infections (SSIs) are a significant cause of morbidity and mortality in low- and middle-income countries, where rates of SSIs can reach 30%. Due to limited access, there is minimal follow up post-operatively. Community health workers (CHWs) have not yet been utilized for surgical patients in most settings. Advancements in telecommunication create an opportunity for mobile health (mHealth) tools to support CHWs. We aim to evaluate the use of mHealth technology to aid CHWs in identification of SSIs and promote referral of patients back to health care facilities.

**Methods and Analysis:** Prospective randomized control trial conducted at Kirehe District Hospital, Rwanda, from November 2017 - November 2018. Patients ≥18 years who undergo cesarean section are eligible. Non-residents of Kirehe District or patients who remain in hospital > 10 days postoperatively will be excluded. Patients will be randomized to one of three arms. For Arm 1, a CHW will visit the patient's home on postoperative day 10 (+/- 3 days) to administer an SSI screening protocol (fever, pain, or purulent drainage) using an electronic tablet. For Arm 2, the CHW will administer the screening protocol over the phone. For both Arms 1 and 2, the CHW will refer patients that respond "yes" to any of the questions to a health facility. For Arm 3, patients will not receive follow-up care. Our primary outcome will be the impact of the mHealth-CHW intervention on the rate of return to care for patients with an SSI.

**Ethics and Dissemination:** The study has received ethical approval from the Rwandan National Ethics Committee and Partners Healthcare. Results will be disseminated to Kirehe District Hospital, Rwanda Ministry of Health, Rwanda Surgical Society, Partners In Health, through conferences, and peer reviewed publications.

**Word Count:** 288/300



For peer review only - http://bmjopen.bmj.com/site/about/guidelines.xhtml

**STRENGTHS AND LIMITATIONS OF STUDY:**

- The greatest strength is that this is a prospective randomized control trial to most effectively evaluate the impact of a mobile health and CHW intervention on return to care following surgery.
- The screening protocol utilized has been previously validated by the study team in this setting.
- The study is well-resourced with significant on the ground logistical support through Partners in Health and the staff at Kirehe District Hospital.
- In addition to assessing the impact on patient return-to-care behaviors, this study will also allow us to describe the feasibility of mHealth and CHW interventions in this setting, beyond surgical interventions.
- Since validating the presence or absence of postoperative infections would interfere with the study aims, we can only compare the proportion of all patients that return to care with confirmed infections and must assume that the infection rates across arms are constant.

For peer review only



For peer review only - http://bmjopen.bmj.com/site/about/guidelines.xhtml

## INTRODUCTION

### Background

Surgical site infections (SSIs) are a major source of morbidity and mortality worldwide and the leading health-care-associated infection in the developing world.[1] The burden is disproportionately felt in low- and middle-income countries (LMICs), and especially by those in Africa where the rates of post-operative SSIs have been documented as high as 30.9%.[2] In these settings, SSIs often develop after patients are discharged home, and geographic and resource barriers prevent patients from routine postoperative follow-up.[3,4] In many LMICs, including Rwanda, follow-up with a surgical care provider after a procedure is not routine. Even when scheduled, rates of follow-up are low. A study from Central African Republic reported only 25% of surgical patients returned for their scheduled 30-day post-operative visit.[5] For patients who develop an SSI, failure to return or a delayed return to care is linked with poor health outcomes including sepsis, need for re-operation, death, and increased healthcare costs.[6]

In many LMICs, community health workers (CHWs) play a major role in delivering household-based care to vulnerable populations who might otherwise be unable to access health facilities.[7,8] Globally, the range of responsibilities of CHWs vary by program, whether polyvalent or topic-focused, such as the maternal and child health CHWs in Rwanda.[9] Regardless of the range, the number of responsibilities is typically high-leading to CHW work overload. Additional activities require extensive pre- or post-service training or provision of activity support aides. Recent advances in telecommunication and increasing access to mobile phones in LMICs create opportunities to use mobile health (mHealth) strategies to support CHWs. In Rwanda, 63% of the population in 2014 reportedly owned a cell phone, with 99% having access to mobile networks.[10] Multiple studies have shown that real-time use of


For peer review only - http://bmjopen.bmj.com/site/about/guidelines.xhtml

mHealth technologies increases adherence to health protocols in rural Africa,[11-15] and also improves the perceived quality of care.[16]

In 2014, members of the study team carried out a pilot study in Haiti that involved CHWs following up with surgical patients once discharged and evaluating their wounds for an SSI.[17] The CHWs used an mHealth application that prompted the CHW to evaluate the wound for certain characteristics pertaining to SSIs as well as to take a photograph of the wound. The CHW's assessment of the wounds were then compared to a surgeon's assessment (using the photograph), and found 85% agreement. In the phase one study precluding this manuscript, over a 4-month period in 2017 (March- July) at KDH, we evaluated C-section patients at post-operative day (POD) 10 (+/- 3 days) and found a 10.3% SSI incidence (results yet to be published). In this study, we draw from lessons learned in the pilot to rigorously explore the use of mHealth-CHW interventions for postoperative follow-up of patients delivering via cesarean sections in rural Rwanda.

**Aims**

The overall study aim is to examine whether CHWs, guided by an mHealth-delivered screening protocol, can improve the identification of SSIs and inform a timely return to care among patients who undergo cesarean sections.

Specific objectives:

- Objective 1: Evaluate the impact of the mHealth-CHW interventions on patients returning to the health center or hospital for a possible SSI.
- Objective 2: To assess the feasibility of an mHealth-CHW intervention for post-operative follow-up.



For peer review only - http://bmjopen.bmj.com/site/about/guidelines.xhtml

After receipt of a voluntary written consent, enrolled patients will be randomly assigned to one of three arms (Figure 1):

- Arm 1– an mHealth-CHW intervention where the CHW visits the patient postoperatively, administers the screening protocol and refers the patient back to care if there is evidence of an SSI;
- Arm 2 – an mHealth-CHW intervention where the CHW calls the patient postoperatively, administers the screening protocol over the phone and refers the patient back to care if there is evidence of an SSI; or
- Arm 3 – standard of care (no routine follow up).

For peer review only

## METHODS AND ANALYSIS

### Study Location

This study will take place between November 2017 – November 2018 at Kirehe District Hospital (KDH)- one of 42 district hospitals in Rwanda. KDH is a Level 1 hospital, with 235 beds, operated by the Rwanda Ministry of Health and supported by the medical non-profit organization Partners In Health (PIH). The hospital serves a catchment area of 368,950 people, primarily residing in rural, outlying villages. KDH performs around 1,400 surgical operations a year, with the majority being cesarean sections (C-sections).[18] Nearly all C-sections are performed by general practitioner (GP) physicians, with occasional surgeries performed by visiting obstetricians.

### Study Population

This study will only include patients undergoing C-section delivery, which are the majority of



For peer review only - http://bmjopen.bmj.com/site/about/guidelines.xhtml

patients undergoing an operation, at KDH. Over 60% of all surgeries performed at Rwandan district hospitals are obstetric related.[19] All patients 18 years or older undergoing a C-section at KDH during a 12 month study enrollment window will be eligible for inclusion. Participants must be residents of Kirehe District. We will exclude patients who remain inpatient after POD 10 as the window for follow up we are interested in would have passed (10 days postoperative +/- 3 days). We will also exclude patients who are residents of Mahama refugee camp in Kirehe as the refugee camp is temporary and the patients are not covered by the existing CHW network.

**The SSI screening protocol**

The study SSI screening protocol will consist of three screening questions, which were developed and validated during Phase 1 of this study. Phase 1 was also carried out at KDH, and the three questions were selected to have the highest sensitivity while maintaining reasonable specificity for diagnosing an SSI. The optimization occurred over a 4-month period in 2017 and included post C-section surgical discharged patients 18 years or older. Patients returned to the hospital for evaluation on POD 10 (+/-3 days) and were evaluated by a general practitioner (GP). A CHW administered a 10-question SSI screening protocol assessing for: 1) increased pain since discharge; 2) fever since discharge, 3) erythema, 4) edema, 5) induration, 6) dehiscence, 7) drainage from the wound, 8) drainage with discoloration, 9) drainage with a foul odor, and 10) drainage with pus (purulent drainage). Using the GP's SSI diagnosis as the gold standard, we identified the following three questions as most sensitive and specific for SSI diagnosis: purulent drainage, pain and fever (Table 1).



For peer review only - http://bmjopen.bmj.com/site/about/guidelines.xhtml

**Table 1.** SSI Screening Protocol

| Question | Answer |
|---|---|
| Have you had a fever since discharge? | Yes/No |
| At the incision, have you had increasing pain? | Yes/ No |
| Any active drainage? | Yes/No |
| - What color is the fluid? | Brown, yellow, green or white / Red, pink, clear |

**Study Interventions**

The study involves two different interventions: use of mHealth and CHWs arms. For both interventions, patients will be screened at POD 10 (+/- 3 days). We selected this window because the majority of SSIs develop between POD 5-10 days and timely identification of SSIs is a critical aspect of the intervention.[20] In Arm 1, a CHW will travel to the patient's home to evaluate the patient. Prior to the visit, the patient will be called to confirm location and time. The hired surgical CHW will contact the local CHW who will guide the surgical CHW to the patient's home. Once at the patient's home, the local CHW will leave, and the surgical CHW will evaluate the patient using the SSI screening protocol administered on an electronic tablet and take a photo of the wound. In Arm 2, a CHW will call the patient on the phone on POD 10 (+/- 3 days). Three attempts will be made to reach the patient. The CHW will administer the screening protocol over the phone, prompted by the tablet application to ask the appropriate questions. For both intervention arms, if the patient answers yes to any of the three questions, the patient will be instructed by the CHW to present to the local health center for evaluation and referral to KDH if necessary. Patients not identified with an SSI will be reminded of proper wound care, warning signs of SSI and to follow-up should there be any change. In Arm 3,



For peer review only - http://bmjopen.bmj.com/site/about/guidelines.xhtml

patients will be given discharge instructions however will not have any contact with a CHW following discharge and therefore will serve as a control group.

**Study Consent, Enrollment, Randomization and Follow-up**

On POD 2, eligible patients will be identified. Study staff will read the consent form (appendix) to the patient in Kinyarwanda and solicit a signed consent. Once the patient is enrolled, there will be no special retention strategies as this will interfere with the overall study outcomes.

At discharge, the enrolled patients will be randomized to one of the three study arms described above. Study staff will prepare study packets, in sealed envelopes numbered consecutively. REDCap application will be used to randomly generate arm assignments to each packet. The assignment is independent of any patient factors, including whether the patient has access to a cell phone or lives in an area with cell phone coverage. In addition to the random arm assignment, the packet will include details on arm-specific follow-up such as follow-up plan for home visits (Arm 1) or phone call date (Arm 2). The packet will also include general discharge instructions including signs of a surgical site infection, how to contact study staff, and how to return to a health center for care or referral to KDH if a SSI is suspected by CHW.

All enrolled patients will be followed for 30 days post-operatively. If a patient is identified as having an SSI, she will be followed up to 90 days to document the progression and treatment of the infection. On POD 30, all patients will be called by a member of the study team to check in to see if they have returned to care. Study participants who return to care will be recorded by the register at the health facility where she presents (health center or hospital), and the study team will have regular check-ins with the register to obtain the list of patients who returned to care. Clinical data from those follow up visits will then be transcribed into REDCap



For peer review only - http://bmjopen.bmj.com/site/about/guidelines.xhtml

for each patient.

**Data Collection and Variables**

All study data will be collected, managed and store using REDCap electronic data capture tools hosted by Brigham and Women's Hospital. REDCap is a secure web application that can support both online or offline data collection for research studies.[21],[22] The REDCap mobile application will be utilized by CHWs to administer the SSI screening protocol. There will be five distinct time points of data collection. Study coordinators will have access to data to evaluate for completeness.

First, upon enrollment, all patients will provide basic demographics, socioeconomic and location data including but not limited to age, occupation, education, household income, insurance, home location, travel distance from the patient's home, patient's home village, cell, sector name, name of local CHW, phone number of the patient, phone number of a family member or a neighbor (in case the patient does not have personal phone), with permissions to call these numbers as part of follow-up. Secondly, on discharge, data collectors will complete a clinical chart review, extracting details on patient's past medical history, intraoperative data (pre-operative antibiotics, wound class, intraoperative complications), and post-operative care. Thirdly, for patients in Arms 1 and Arms 2, we will collect the responses of the SSI screening protocol. The CHW will click on the patient's ID number in REDCap, and the application will prompt the CHW to ask the three SSI screening protocol questions. The CHW will answer the questions on the tablet and the data will be stored. The fourth round will include the CHW separately collecting data on process indicators related to the implementation of the intervention. For Arm 1, these indicators will include: ability to visit the patient on the scheduled date, ability



For peer review only - http://bmjopen.bmj.com/site/about/guidelines.xhtml

to find the local CHW and the patient's home, travel time, presence of the patient at time of visit, willingness of the patient to allow CHW into home, patient compliance with the SSI screening protocol, if the patient allowed the CHW to perform an examination/ take a photo of the wound, and if there were any technical difficulties with the tablet or software. For Arm 2, these indicators will include: whether the patient was reached by phone, how many attempts were made, which number was called and who answered, total call time, and whether patient allowed the CHW to administer the SSI screening protocol. Finally, we will track the patient's return to care within 30 days post-operatively using a register posted at each of the 16 district health centers where staff can record any study patients who present to that location for care. The head of maternity at each health center will be a point person for this follow up register. The study coordinator will call each point person to check if a C-section patient showed up at any health center. If so, the study coordinator will visit the health centers that patients returned to. During the visit, the study coordinator will refer to the follow-up register to record into REDCap which date the patient returned, wound status, diagnosis, treatment provided, and if they were referred to KDH for further care. There will be a similar patient tracker log in the maternity ward reception at KDH to document patients referred to the hospital. This log will be completed by the reception nurse who will notify the study data collector who will input to REDCap. Finally, all patients with phone numbers provided will be called on POD 30 to inquire about any re-admissions or visits to other healthcare facilities. Study staff will extract from the clinical chart the presence of an SSI, severity, treatment obtained, need for operative intervention, hospitalization, and/ or complications.

**Analyses**



For peer review only - http://bmjopen.bmj.com/site/about/guidelines.xhtml

All analyses will be completed as intention to treat. For objective 1, the primary outcome is whether a patient returns to care at a health center or district hospital with a provider-confirmed SSI. We will compare the proportion of patients who returned for follow-up with an SSI in Arms 1 and 2 to Arm 3 using a two-sided, two-sample test of proportions at the $\alpha=0.05$ significance level. The analyses assume that the rates of true SSIs are constant across the three arms, but that the proportion of these infections that return to care will vary across the study arms as a result of the intervention. We have purposely chosen not to trace patients to establish their true SSI status, as this would interfere with care seeking behavior. However, we will perform a sensitivity analysis (changing the null hypothesis from $p_1=p_2$ to $p_1=kp_2$, where $k$ reflects differences in SSI rates) to determine under what range of SSI rates the results are still valid. As a secondary outcome for objective 1, we will look at time to return-to-care for patients with SSI dichotomized as within 15 PODs or more than 15 PODs. We will use a logistic regression model to assess the impact of study arm on timely return to care, controlling for potential confounders collected at enrollment. For objective 2, we will assess the implementation feasibility of the CHW-mHealth intervention by quantifying intervention indicators. For each indicator, we will report the percent of eligible encounters for which that step was successfully completed, and will categorize a specific component as feasible if at least 85% of eligible counters have that step completed. For Arms 1 and 2, we will calculate a comprehensive feasibility measure that will assess the percent of encounters that successfully implemented the full intervention, which we aim to achieve with at least 85% of patient encounters.

**Power calculation**

Over the 12-month study period, we expect 78 patients/month or 1092 patients total to be eligible



For peer review only - http://bmjopen.bmj.com/site/about/guidelines.xhtml

for inclusion. Assuming a 1-1-1 randomization, 26 patients/month or 364 patients total will be randomized to each arm (Table 2).

**Table 2.** Sample Size Calculation

| | **Arm 1:** Home visit + Protocol | **Arm 2:** Phone call + Protocol | **Arm 3:** Standard of care |
|---|---|---|---|
| Total Patients | 364 | 364 | 364 |
| Anticipated SSIs | 55 | 55 | 55 |
| Hypothesized patients to return with SSIs | 44 (80%) | 44 (80%) | 22 (40%) |
| Overall hypothesized proportion that will return with SSI | 0.12 | 0.12 | 0.06 |

We assume a constant SSI rate across the three arms of 15% (based on data from preliminary chart reviews prior to this study, and prior to the first phase of this study which identified the 10.3% prevalence over a seven-month enrollment window). We assume more patients with SSIs will return to care in Arms 1 and 2 compared to Arm 3 (80% of SSIs in Arms 1 and 2 compared to 40% in Arm 3). This corresponds to an overall return to care rate of 12% in Arms 1 and 2 and 6% in Arm 3. We would have an 81% power to detect a difference between the proportion of patients that returned with an SSI in Arms 1 and 2 (12%) as compared to Arm 3 (6%) with a two-sided test at the $\alpha$=0.05 significance level.

**Patient and Public Involvement**

Patients and/or the public were not involved with the development of the research question or study design. The results of the study however will be disseminated at a community event at the hospital following the completion of the trial.



For peer review only - http://bmjopen.bmj.com/site/about/guidelines.xhtml

## ETHICS AND DISSEMINATION:

Study participants will be informed on the intent of the study, potential benefits and risks of their enrollment, and how these will be minimized. Those who wish to enroll will be informed of their right to withdraw throughout the study period. All data collectors will sign confidentiality training and agreements; study coordinators and CHWs. Risks to privacy will be minimized by having all mobile devices and computers password protected. Data will be stored on HIPAA-compliant servers, and data will be de-identified prior to any analysis.

### Benefits, Risks and Limitations

The study does not alter the standard of care in any way and therefore there is minimal to no increased risk to the patient. Participants will likely benefit from this study in that the intervention we hypothesize will lead to a timelier diagnosis of SSI and will encourage patients to return to care, which is likely to correlate with improved health outcomes. However, one limitation of this study is that we do not measure health outcomes directly. Patients enrolled in both Arms 1 and 2 will have additional contact with a health care provider (CHW) beyond the current standard of care. While not all participants may need this earlier screening, as not all will have surgical complications, the risks and discomforts associated with the screening are minimal. Given that patients will be randomized to all three arms, there is a risk of cross contamination between patients from the same village. However, with our total sample size of 1200 patients, and that Kirehe District has approximately 612 villages with the population relatively evenly distributed, we do not expect more than 2-5 women per village to be enrolled.  Since enrollment will be over 12 months, we expected that this contamination bias will be minimal.



For peer review only - http://bmjopen.bmj.com/site/about/guidelines.xhtml

On a systems level, this study will benefit the local providers and research staff to understand whether CHWs can be used in this capacity for postoperative follow-up. If we find that routine follow up of patients with a CHW (either by phone or in-person visits) leads to a statistically significant higher identification of patients with an SSI, we will then be able to advocate for the use of CHWs for postoperative patients as that currently is not the standard. Further, given the relationship that the study staff has with the CHW coordinator for Kirehe District, KDH, as well as the Ministry of Health, it could lead to a new standard of care for all patients to have regular follow up after cesarean section. In addition, this study tracks feasibility indicators, which will inform broader conversations about whether such follow-up is possible in this and similar contexts; this is particularly novel for the Arm 2, given that no programs have used phone calls for post-operative follow-up in the rural areas in the region.

A potential risk will be decreasing the likelihood of a patient return to care when needed under the mHealth-CHW interventions. It is possible that the CHW will give the wrong SSI diagnosis or that a patient may delay return to care because of an expected visit from a CHW. This risk is moderate and will be monitored by a Data and Safety Monitoring Board (DSMB). Finally, a potential risk would be a breach in confidentiality, resulting in the disclosure of patient information. This risk is considered minimal as unique codes will be used in place of participant names throughout the study. Only PIs and study coordinators will have access to the final de-identified database.

**Data and Safety Monitoring Board (DSMB)**

The DSMB will be designated to oversee the safety and effectiveness of the study. This committee will include one global surgery expert, one Rwandan health practitioner and one



For peer review only - http://bmjopen.bmj.com/site/about/guidelines.xhtml

statistician. Meetings of the DSMB will be held twice – once at the start of the study and 6 months after the start of Phase 2. At the first meeting the DSMB will discuss the protocol, suggest modifications, and establish guidelines to study monitoring by the Board.

At the second meeting, we will present the DSMB an interim analysis report, which will compare rates of return between the three study arms and include a list of adverse events of this study, if any. We anticipate ½ of the total cohort of patients will be included in this interim analysis. If the proportion who have returned in Arms 1 and 2 is significantly lower compared to standard of care, then the study will be stopped or one study arm will be dropped. Further, if there are significantly more complex cases at return (higher rates of readmission or reoperation) in Arms 1 or 2, then the study will be stopped or one study arm will be dropped. The outcome of the DSMB review will be summarized in a letter to the IRBs of all participating institutions. A recommendation by the DSMB to terminate the study would be communicated to the NIH Director, who will then accept or decline the recommendation.

**Ethics Approvals**

The study has received IRB approval both in the United States and in Rwanda. IRB approval in the United States has been achieved through Partners Healthcare (2016P001943/MGH). The Rwanda National Ethics Committee has reviewed and approved the study (848/RNEC/2016). Any proposed protocol amendments would undergo review and approvals by IRBs before further implementation.

**Dissemination**

Results will be disseminated to the staff at Kirehe District Hospital, the Rwanda Ministry of



For peer review only - http://bmjopen.bmj.com/site/about/guidelines.xhtml

Health, including the electronic Health and CHW departments, the Rwanda Surgical Society, and PIH. Results will also be disseminated at regional and international conferences and via peer reviewed publications.

For peer review only



For peer review only - http://bmjopen.bmj.com/site/about/guidelines.xhtml

**AUTHORS CONTRIBUTIONS:** BHG, RR and FK are the three primary investigators for the study, instigated the original idea for the study, developed the funding proposal, and applied for funding. EG, CH, AM, EN, GN advised the study objectives and scientific content of this study. KAS, TN and RK are study coordinators and contributed to writing of the protocol. MG contributed to writing of the protocol and Figures/Tables. All authors read and commented on drafts, and approved the final version.

**NO AUTHORS HAD COMPETING INTERESTS**

**FUNDING/SPONSOR:** This work was supported by The National Institute of Health grant number NIH Grant 1R21EB022369 – 01. The funders have no role in the study design, collection, management, analysis, or interpretation of results.  Contact info: Ruthann Rand, Grants Management Officer, email: Rudy.Rand@nih.hhs.gov

For peer review only

**Figure Legend**

Figure 1. Study Design

For peer review only



For peer review only - http://bmjopen.bmj.com/site/about/guidelines.xhtml

## REFERENCES


1 Allegranzi B, Bagheri Nejad S, Combescure C, *et al.* Burden of endemic health-care-associated infection in developing countries: systematic review and meta-analysis. *Lancet (London, England)* 2011;**377**:228–41. doi:10.1016/S0140-6736(10)61458-4

2 Nejad SB, Allegranzi B, Syed S, *et al.* Health-care-associated infection in Africa: a systematic review. *Bull World Health Organ* 2011;**89**:757–65. doi:10.2471/BLT.11.088179

3 Grimes CE, Bowman KG, Dodgion CM, *et al.* Systematic Review of Barriers to Surgical Care in Low-Income and Middle-Income Countries. *World J Surg* 2011;**35**:941–50. doi:10.1007/s00268-011-1010-1

4 Hsia RY, Mbembati NA, Macfarlane S, *et al.* Access to emergency and surgical care in sub-Saharan Africa: the infrastructure gap. *Health Policy Plan* 2012;**27**:234–44. doi:10.1093/heapol/czr023

5 Bercion R, Gaudeuille A, Mapouka PA, *et al.* [Surgical site infection survey in the orthopaedic surgery department of the "Hôpital communautaire de Bangui," Central African Republic]. *Bull Soc Pathol Exot* 2007;**100**:197–200.http://www.ncbi.nlm.nih.gov/pubmed/17824315 (accessed 10 Jun 2017).

6 Gottrup F, Melling A, Hollander D. An overview of surgical site infections: aetiology, incidence and risk factors. *EWMA J* Published Online First: 2005.http://www.worldwidewounds.com/2005/september/Gottrup/Surgical-Site-Infections-Overview.html (accessed 13 Jun 2017).

7 Dillip A, Kimatta S, Embrey M, *et al.* Can formalizing links among community health workers, accredited drug dispensing outlet dispensers, and health facility staff increase their collaboration to improve prompt access to maternal and child care? A qualitative study in Tanzania. *BMC Health Serv Res* 2017;**17**:416. doi:10.1186/s12913-017-2382-1

8 Asiimwe S, Ross JM, Arinaitwe A, *et al.* Expanding HIV testing and linkage to care in southwestern Uganda with community health extension workers. *J Int AIDS Soc* 2017;**20**:80–7. doi:10.7448/IAS.20.5.21633

9 Haver J, Brieger W, Zoungrana J, *et al.* Experiences engaging community health workers to provide maternal and newborn health services: Implementation of four programs. *Int J Gynecol Obstet* 2015;**130**:S32–9. doi:10.1016/j.ijgo.2015.03.006

10 *Statistical YearBook 2014 | National Institute of Statistics Rwanda*. Kigali: 2014. http://statistics.gov.rw/publication/statistical-yearbook-2016 (accessed 10 Jul 2017).

11 Mitchell M, Hedt-Gauthier B. Using electronic technology to improve clinical care–results from a before-after cluster trial to evaluate assessment and classification of sick children. *BMC Med* Published Online First: 2013.https://bmcmedinformdecismak.biomedcentral.com/articles/10.1186/1472-6947-13-95 (accessed 13 Jun 2017).

12 Ngabo F, Nguimfack J, Nwaigwe F, *et al.* Designing and Implementing an Innovative SMS-based alert system (RapidSMS-MCH) to monitor pregnancy and reduce maternal and child deaths in Rwanda. *Pan Afr Med J* 2012;**13**:31.http://www.ncbi.nlm.nih.gov/pubmed/23330022 (accessed 25 Aug 2017).

13 Elbur AI, MA Y, ElSayed ASA, *et al.* Post-discharge surveillance of wound infections by telephone calls method in a Sudanese Teaching Hospital. *J Infect Public Health* 2013;**6**:339–46. doi:10.1016/j.jiph.2013.04.005




For peer review only - http://bmjopen.bmj.com/site/about/guidelines.xhtml

14 Nguhuni B, De Nardo P, Gentilotti E, *et al.* Reliability and validity of using telephone calls for post-discharge surveillance of surgical site infection following caesarean section at a tertiary hospital in Tanzania. *Antimicrob Resist Infect Control* 2017;**6**:43. doi:10.1186/s13756-017-0205-0
15 Matousek A, Paik K, Winkler E, *et al.* Community health workers and smartphones for the detection of surgical site infections in rural Haiti: a pilot study. *Lancet* 2015;**385**:S47. doi:10.1016/S0140-6736(15)60842-X
16 Mitchell M, Getchell M, Nkaka M. Perceived improvement in integrated management of childhood illness implementation through use of mobile technology: qualitative evidence from a pilot study in. *J Heal* Published Online First: 2012.http://www.tandfonline.com/doi/abs/10.1080/10810730.2011.649105 (accessed 13 Jun 2017).
17 Matousek A, Addington S, Paik K. Community Health Workers for Surgery: A Pilot Study of an mHealth Application for the Early Detection of Surgical Site Infection in Rural Haiti. *J* Published Online First: 2014.https://www.journalacs.org/article/S1072-7515(14)00676-0/fulltext (accessed 13 Jun 2017).
18 Muhirwa E, Habiyakare C, Hedt-Gauthier BL, *et al.* Non-Obstetric Surgical Care at Three Rural District Hospitals in Rwanda: More Human Capacity and Surgical Equipment May Increase Operative Care. *World J Surg* 2016;**40**:2109–16. doi:10.1007/s00268-016-3515-0
19 Petroze RT, Nzayisenga a, Rusanganwa V, *et al.* Comprehensive national analysis of emergency and essential surgical capacity in Rwanda. *Br J Surg* 2012;**99**:436–43. doi:10.1002/bjs.7816
20 Health NCC for W and C. Surgical Site Infection: Prevention and Treatment of Surgical Site Infection. London: : RCOG Press 2008. https://www.ncbi.nlm.nih.gov/books/NBK53724/
21 Klipin M, Mare I, Hazelhurst S, *et al.* The process of installing REDCap, a web based database supporting biomedical research: the first year. *Appl Clin Inform* 2014;**5**:916–29. doi:10.4338/ACI-2014-06-CR-0054
22 Harris PA, Taylor R, Thielke R, *et al.* Research electronic data capture (REDCap)--a metadata-driven methodology and workflow process for providing translational research informatics support. *J Biomed Inform* 2009;**42**:377–81. doi:10.1016/j.jbi.2008.08.010

For peer review only



For peer review only - http://bmjopen.bmj.com/site/about/guidelines.xhtml

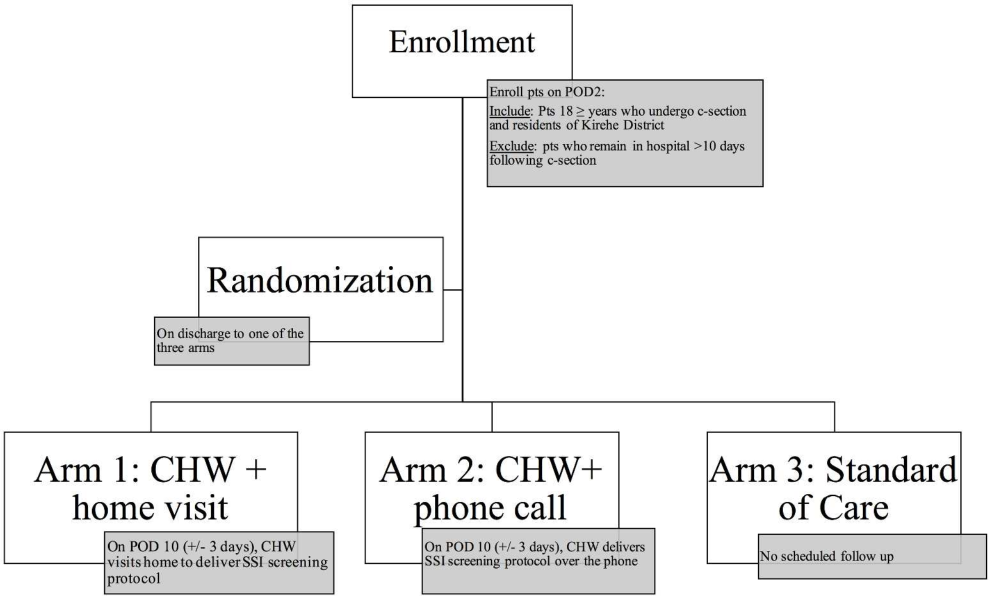


Study Design

303x181mm (300 x 300 DPI)

For peer review only - http://bmjopen.bmj.com/site/about/guidelines.xhtml

**Appendix.** Consent Form (English Version)

## INFORMED CONSENT FORM

This Informed Consent Form is for women 18 years of age and above who attend Kirehe District Hospital and receive cesarean section surgery. You are invited to participate in research on follow up of patients with surgical site infections post operation using mobile phones.

**The title of our research project is**: Using mHealth technology to identify and refer surgical site infections in Rwanda

**Principal Investigators (PIs):**
Robert Riviello
Rwanda Human Resources for Health Program, Harvard Medical School, Brigham and Women's Hospital
robertriviello@gmail.com +250783002502

Bethany Hedt-Gauthier
Partners in Health and Harvard Medical School
bethhedt@gmail.com +18572251945

Fred Kateera
Partners In Health
fkateera@partnersinhealth.onmicrosoft.com +250784684871

This consent form will give you the information you will need to understand why this study is being done and why you are being invited to participate. It also describes what you will need to do to participate. We encourage you to ask questions at any time. If you decide to participate, you will be asked to sign this form and I will keep it as a record of your agreement to participate. I will gladly provide you with a copy of this form to keep for your records upon your request.

**PURPOSE AND BACKGROUND**

Surgical site infections (SSI) represent a major source of morbidity and mortality worldwide and are disproportionately felt in low- and middle-income countries. You are invited to participate in a research study to assess the impact of the mobileHealth-supported delivery of the screening protocol by surgical CHWs on the rate of return to care of patients with SSI ten days post-operative. For patients who return, we will assess the severity of SSI at return to care. We aim to investigate timely and appropriate return to care of patients with SSIs in Rwanda, improving patient outcomes and reducing healthcare costs.

**PROCEDURES**

If you agree to participate in the study, you will be randomized into one of three study arms – Arm 1: home visit from the sCHW with screening using the mHealth tool; Arm 2: screening by sCHW over the phone using the mHealth tool; and Arm 3: standard of care, with no special contact from the sCHW or interaction with the mHealth tool. You will be instructed to return to

For peer review only

your local health center as soon as any of the signs of infection present. The study team will record basic demographic and clinical data.

If you are randomized into Arm 1 or 2, we will ask for addresses/phone numbers and availability to allow for follow-up by the sCHW. If you are randomized to Arm 1, sCHWs will visit you at ten post-operative days (±3 days) at the address provided. The sCHW will be assisted by a local village CHW to identify your home. Once there, the sCHW will administer the SSI screening protocol. A picture of your wound and GPS coordinates for your location will be taken. If you are randomized to Arm 2, you will be called by the sCHW on the tenth post-operative day (±3 days). The sCHW will administer the SSI screening protocol over the phone, prompted by the mHealth tool to ask the appropriate questions. If you are identified as having an SSI, the sCHW will ask you to go to your health center for care and from there you can be referred to KDH if necessary. If you are not identified to have an SSI, you will be reminded of the warning signs and follow-up instructions.

If you are randomized to Arm 3, you will receive standard of care, which is information upon your discharge about the signs of SSI. You will not receive any follow up from the sCHWs. You will be advised to return to your regional health center if any of the signs of an SSI do occur.

**PARTICIPANT SELECTION**
We are inviting all adults of 18 years and above who attend Kirehe District Hospital and receive cesarean section surgery to participate in this study.

**RISKS**
You will receive standard of care advice on surgical follow-up and when to return to care. If you are randomized into an arm where you have contact with a sCHW (Arms 1 and 2), you will be referred back to care if evidence of an SSI is present or will otherwise be reminded of advice on when to return to care. It is possible that the sCHW will give the wrong SSI diagnosis or that a patient may delay return to care because of an expected visit from an sCHW. This risk is moderate as the SSI screening protocol will have been tested for accuracy. However, this risk will be monitored.

**BENEFITS**
If you are randomized to Arms 1 or 2, you will have additional contact with a health care provider (sCHW) beyond the standard of care, which may lead to a more timely diagnosis of SSI. This may lead to an earlier presentation to care for appropriate treatment. You may also benefit from decreased barriers to follow-up care. Your participation may also help design quality improvement interventions that have the potential to directly affect the quality and efficiency of surgical care at KDH and other hospitals in Rwanda.

**EXTENT OF CONFIDENTIALITY**
Participation in research may involve a loss of privacy; however, your records will be handled as confidentially as possible. We will not be sharing the identity or information of those participating in the research. Information we collect from this research will be kept confidential and no one but the study staff will be able to see it. Your name will not be used in any written reports or publications that result from this research. Any information about you will have a

For peer review only

unique study number on it instead of your name. Only the study staff will know the number and we will lock that information up with a lock and key. Data will be kept for three years after the study is complete and then destroyed, per United States federal regulations.

**PAYMENT**
You will not receive any monetary compensation for participation in this study.

**QUESTIONS**
If you have any questions or concerns about your participation in this study, you should first contact the principal investigators at +250784684871 or bethhedt@gmail.com or robertriviello@gmail.com. If you have questions about your rights as a research participant, you may contact the Partners Healthcare Institutional Review Board (IRB), which is concerned with the protection of volunteers in research projects. You may reach the board office by calling +1 (617) 424-4100, or by emailing IRB@partners.org. Responses will be provided in one business day.

**PARTICIPATION IS VOLUNTARY**
You do not have to participate in this study if you do not want to. If you volunteer to be in this study, you may withdraw from it at any time without consequences of any kind or loss of benefits to which you are otherwise entitled. Whether you choose to participate or not does not impact the standard of care you receive from Kirehe District Hospital.

**DOCUMENTATION OF CONSENT**
I have read the information in this Informed Consent Form, or it has been read to me. Its general purposes, the particulars of involvement and possible risks, including the questions I have asked, have been explained to my satisfaction. I understand the information in this form and I have decided that I will participate in the research project described above. I understand I can withdraw at any time.

For peer review only

____________________________ ____________________________________
**Printed Name** of Study Participant **Signature** of Study Participant Date

____________________________ ___________
Signature of Person Obtaining Consent Date

**If the participant cannot read or write:**

I have witnessed the accurate reading of this Informed Consent Form to the potential participant, and the individual has had the opportunity to ask questions. I confirm that the individual has given consent freely.

**Print name** of witness______________________ **AND** **Thumb print** of participant

**Signature** of witness ______________________

Date ______________________

For peer review only

STANDARD PROTOCOL ITEMS: RECOMMENDATIONS FOR INTERVENTIONAL TRIALS

SPIRIT 2013 Checklist: Recommended items to address in a clinical trial protocol and related documents*

| Section/item | ItemNo/ Pg No | Description |
|---|---|---|
| **Administrative information** | | |
| Title | 1/ 1 | Descriptive title identifying the study design, population, interventions, and, if applicable, trial acronym |
| Trial registration | 2/ 1 | Trial identifier and registry name. If not yet registered, name of intended registry |
| | 2b/ yes, per below | All items from the World Health Organization Trial Registration Data Set |
| Protocol version | 3/ 1 | Date and version identifier |
| Funding | 4/17 | Sources and types of financial, material, and other support |
| Roles and responsibilities | 5a/ 1,17 | Names, affiliations, and roles of protocol contributors |
| | 5b/ 17 | Name and contact information for the trial sponsor |
| | 5c/ 17 | Role of study sponsor and funders, if any, in study design; collection, management, analysis, and interpretation of data; writing of the report; and the decision to submit the report for publication, including whether they will have ultimate authority over any of these activities |
| | 5d /14,15 | Composition, roles, and responsibilities of the coordinating centre, steering committee, endpoint adjudication committee, data management team, and other individuals or groups overseeing the trial, if applicable (see Item 21a for data monitoring committee) |
| **Introduction** | | |
| Background and rationale | 6a/ 4 | Description of research question and justification for undertaking the trial, including summary of relevant studies (published and unpublished) examining benefits and harms for each intervention |
| | 6b/ 6 | Explanation for choice of comparators |
| Objectives | 7/ 5,6 | Specific objectives or hypotheses |

For peer review only

| | | |
|---|---|---|
| Trial design | 8/ 8 | Description of trial design including type of trial (eg, parallel group, crossover, factorial, single group), allocation ratio, and framework (eg, superiority, equivalence, noninferiority, exploratory) |

**Methods: Participants, interventions, and outcomes**

| | | |
|---|---|---|
| Study setting | 9/ 6 | Description of study settings (eg, community clinic, academic hospital) and list of countries where data will be collected. Reference to where list of study sites can be obtained |
| Eligibility criteria | 10/ 7 | Inclusion and exclusion criteria for participants. If applicable, eligibility criteria for study centres and individuals who will perform the interventions (eg, surgeons, psychotherapists) |
| Interventions | 11a/ 8 | Interventions for each group with sufficient detail to allow replication, including how and when they will be administered |
| | 11b/ 14,15 | Criteria for discontinuing or modifying allocated interventions for a given trial participant (eg, drug dose change in response to harms, participant request, or improving/worsening disease) |
| | 11c/ 11 | Strategies to improve adherence to intervention protocols, and any procedures for monitoring adherence (eg, drug tablet return, laboratory tests) |
| | 11d/ NA | Relevant concomitant care and interventions that are permitted or prohibited during the trial |
| Outcomes | 12/ 12 | Primary, secondary, and other outcomes, including the specific measurement variable (eg, systolic blood pressure), analysis metric (eg, change from baseline, final value, time to event), method of aggregation (eg, median, proportion), and time point for each outcome. Explanation of the clinical relevance of chosen efficacy and harm outcomes is strongly recommended |
| Participant timeline | 13/ 9,10 | Time schedule of enrolment, interventions (including any run-ins and washouts), assessments, and visits for participants. A schematic diagram is highly recommended (see Figure) |
| Sample size | 14/ 13 | Estimated number of participants needed to achieve study objectives and how it was determined, including clinical and statistical assumptions supporting any sample size calculations |
| Recruitment | 15/ NA | Strategies for achieving adequate participant enrolment to reach target sample size |

**Methods: Assignment of interventions (for controlled trials)**

Allocation:

| | | |
|---|---|---|
| Sequence generation | 16a/ 9 | Method of generating the allocation sequence (eg, computer-generated random numbers), and list of any factors for stratification. To reduce predictability of a random sequence, details of any planned restriction (eg, blocking) should be provided in a separate document that is unavailable to those who enrol participants or assign interventions |
| Allocation concealment mechanism | 16b/ 9 | Mechanism of implementing the allocation sequence (eg, central telephone; sequentially numbered, opaque, sealed envelopes), describing any steps to conceal the sequence until interventions are assigned |
| Implementation | 16c/ 9 | Who will generate the allocation sequence, who will enrol participants, and who will assign participants to interventions |
| Blinding (masking) | 17a/ NA | Who will be blinded after assignment to interventions (eg, trial participants, care providers, outcome assessors, data analysts), and how |
| | 17b/ NA | If blinded, circumstances under which unblinding is permissible, and procedure for revealing a participant's allocated intervention during the trial |
| **Methods: Data collection, management, and analysis** | | |
| Data collection methods | 18a/ 10,11 | Plans for assessment and collection of outcome, baseline, and other trial data, including any related processes to promote data quality (eg, duplicate measurements, training of assessors) and a description of study instruments (eg, questionnaires, laboratory tests) along with their reliability and validity, if known. Reference to where data collection forms can be found, if not in the protocol |
| | 18b/ 11 | Plans to promote participant retention and complete follow-up, including list of any outcome data to be collected for participants who discontinue or deviate from intervention protocols |
| Data management | 19/ 13,14 | Plans for data entry, coding, security, and storage, including any related processes to promote data quality (eg, double data entry; range checks for data values). Reference to where details of data management procedures can be found, if not in the protocol |
| Statistical methods | 20a/ 11,12 | Statistical methods for analysing primary and secondary outcomes. Reference to where other details of the statistical analysis plan can be found, if not in the protocol |
| | 20b/ 11,12 | Methods for any additional analyses (eg, subgroup and adjusted analyses) |

| | | |
|---|---|---|
| | 20c/ 11,12 | Definition of analysis population relating to protocol non-adherence (eg, as randomised analysis), and any statistical methods to handle missing data (eg, multiple imputation) |
| **Methods: Monitoring** | | |
| Data monitoring | 21a/ 14,15 | Composition of data monitoring committee (DMC); summary of its role and reporting structure; statement of whether it is independent from the sponsor and competing interests; and reference to where further details about its charter can be found, if not in the protocol. Alternatively, an explanation of why a DMC is not needed |
| | 21b/ 15 | Description of any interim analyses and stopping guidelines, including who will have access to these interim results and make the final decision to terminate the trial |
| Harms | 22/ 14 | Plans for collecting, assessing, reporting, and managing solicited and spontaneously reported adverse events and other unintended effects of trial interventions or trial conduct |
| Auditing | 23/ NA | Frequency and procedures for auditing trial conduct, if any, and whether the process will be independent from investigators and the sponsor |
| **Ethics and dissemination** | | |
| Research ethics approval | 24/ 15 | Plans for seeking research ethics committee/institutional review board (REC/IRB) approval |
| Protocol amendments | 25/ 15 | Plans for communicating important protocol modifications (eg, changes to eligibility criteria, outcomes, analyses) to relevant parties (eg, investigators, REC/IRBs, trial participants, trial registries, journals, regulators) |
| Consent or assent | 26a/ 9 | Who will obtain informed consent or assent from potential trial participants or authorised surrogates, and how (see Item 32) |
| | 26b/ 9 | Additional consent provisions for collection and use of participant data and biological specimens in ancillary studies, if applicable |
| Confidentiality | 27/ 13,14 | How personal information about potential and enrolled participants will be collected, shared, and maintained in order to protect confidentiality before, during, and after the trial |
| Declaration of interests | 28/ 17 | Financial and other competing interests for principal investigators for the overall trial and each study site |
| Access to data | 29/ 14 | Statement of who will have access to the final trial dataset, and disclosure of contractual agreements that limit such access for investigators |

review only

| | | |
|---|---|---|
| Ancillary and post-trial care | 30/ NA | Provisions, if any, for ancillary and post-trial care, and for compensation to those who suffer harm from trial participation |
| Dissemination policy | 31a/ 16 | Plans for investigators and sponsor to communicate trial results to participants, healthcare professionals, the public, and other relevant groups (eg, via publication, reporting in results databases, or other data sharing arrangements), including any publication restrictions |
| | 31b/ NA | Authorship eligibility guidelines and any intended use of professional writers |
| | 31c/ NA | Plans, if any, for granting public access to the full protocol, participant-level dataset, and statistical code |
| **Appendices** | | |
| Informed consent materials | 32/ 19,20 | Model consent form and other related documentation given to participants and authorised surrogates |
| Biological specimens | 33/ NA | Plans for collection, laboratory evaluation, and storage of biological specimens for genetic or molecular analysis in the current trial and for future use in ancillary studies, if applicable |

*It is strongly recommended that this checklist be read in conjunction with the SPIRIT 2013 Explanation & Elaboration for important clarification on the items. Amendments to the protocol should be tracked and dated. The SPIRIT checklist is copyrighted by the SPIRIT Group under the Creative Commons "Attribution-NonCommercial-NoDerivs 3.0 Unported" license.

For peer review only
